# A Hybrid Genetic Linkage Map of Two Ecologically and Morphologically Divergent Midas Cichlid Fishes (*Amphilophus* spp.) Obtained by Massively Parallel DNA Sequencing (ddRADSeq)

**DOI:** 10.1534/g3.112.003897

**Published:** 2013-01-01

**Authors:** Hans Recknagel, Kathryn R. Elmer, Axel Meyer

**Affiliations:** Lehrstuhl für Zoologie und Evolutionsbiologie, Department of Biology, University of Konstanz, 78457 Konstanz, Germany

**Keywords:** Midas cichlid, double-digest RADSeq, synteny, segregation distortion, RAD markers, mutation rate

## Abstract

Cichlid fishes are an excellent model system for studying speciation and the formation of adaptive radiations because of their tremendous species richness and astonishing phenotypic diversity. Most research has focused on African rift lake fishes, although Neotropical cichlid species display much variability as well. Almost one dozen species of the Midas cichlid species complex (*Amphilophus* spp.) have been described so far and have formed repeated adaptive radiations in several Nicaraguan crater lakes. Here we apply double-digest restriction-site associated DNA sequencing to obtain a high-density linkage map of an interspecific cross between the benthic *Amphilophus astorquii* and the limnetic *Amphilophus zaliosus*, which are sympatric species endemic to Crater Lake Apoyo, Nicaragua. A total of 755 RAD markers were genotyped in 343 F_2_ hybrids. The map resolved 25 linkage groups and spans a total distance of 1427 cM with an average marker spacing distance of 1.95 cM, almost matching the total number of chromosomes (*n* = 24) in these species. Regions of segregation distortion were identified in five linkage groups. Based on the pedigree of parents to F_2_ offspring, we calculated a genome-wide mutation rate of 6.6 × 10^−8^ mutations per nucleotide per generation. This genetic map will facilitate the mapping of ecomorphologically relevant adaptive traits in the repeated phenotypes that evolved within the Midas cichlid lineage and, as the first linkage map of a Neotropical cichlid, facilitate comparative genomic analyses between African cichlids, Neotropical cichlids and other teleost fishes.

Cichlid fishes are a well-known evolutionary model system for studying the mechanisms of speciation and the formation of adaptive radiations. African Rift Lake cichlids in particular display an astonishing variability in phenotypes and comprise an enormous number of species, which makes Cichlidae one of the most species-rich families in vertebrates (Fryer and Iles 1972; Salzburger and Meyer 2004; Berra 2007). Because of this mega-diversity, Rift Lake African cichlids have been the primary focus of genomic research in this family to date (reviewed in Fan *et al.* 2012), but the >60 million year divergent (Matschiner *et al.* 2011) Neotropical lineage of cichlids is pertinent if we are to understand cichlid diversity at the family level and in a phylogenetic context. For example, the Neotropical Midas cichlid lineage alone features some of the key traits that characterize African cichlid fish diversity: polymorphisms in color, jaw, lip, tooth, and body shape (Meyer 1990a,b; Elmer *et al.* 2010b).

Because of their geographic distribution, which includes the two Nicaraguan Great Lakes and the independent colonizations of at least eight crater lakes of Nicaragua, Midas cichlid communities comprise repeatedly evolved populations at different evolutionary stages of local adaptation and speciation (Wilson *et al.* 2000; Barluenga and Meyer 2010). The two oldest crater lakes house most of the described species (9 of 11). Interestingly, some of the younger crater lakes harbor notably different ecotypes in sympatry that are genetically not differentiated from each other (*e.g.*, Elmer *et al.* 2010c). What makes this system so interesting is that similar morphologies, such as elongated body shapes or hypertrophied lips, have evolved independently and repeatedly in different crater lakes (Elmer *et al.* 2010b; Manousaki *et al.* 2013). These two key aspects, (1) the crater lakes as “natural experiments” that contain replicates of cichlid evolution at various stages of diversification, and (2) the ecomorphological variability that evolved in different environments in parallel, facilitates the investigation of mechanisms and discovery of principles of how and which genetic material underlies adaptation and the formation of new species (Elmer and Meyer 2011). For example, knowing the genetics that lead to phenotypic changes via quantitative trait loci (QTL) mapping and association studies in closely and more distantly related species will ultimately help to elucidate how selection has led to the astonishing biodiversity of African and Neotropical cichlids observed today.

In at least two instances, Midas cichlid radiations have formed repeatedly in Nicaraguan crater lakes; specifically the independent formation of several high-bodied endemic species that occupy benthic niche and one elongated limnetic species each in two lakes (Vivas and McKaye 2001; Barluenga *et al.* 2006; Elmer *et al.* 2010b). Apart from body shape, limnetic and benthic species also differ in diet, pharyngeal jaw morphology, behavior, and coloration (Barlow and Munsey 1976; Baylis 1976a,b; Vivas and McKaye 2001; Barluenga *et al.* 2006; Elmer *et al.* 2010b; Lehtonen *et al.* 2011). In Lake Apoyo, which is the oldest of all Nicaraguan crater lakes at 22,000 years of age, speciation into these two distinct ecotypes has occurred *in situ* in the form of sympatric species by ecological speciation (Barluenga *et al.* 2006). Evidence suggests that benthic-limnetic divergence in Xiloa was also in sympatry (Kautt *et al.* 2012). The linkage mapping analysis in this study is based on a cross of the sympatrically occurring limnetic *Amphilophus zaliosus* and benthic *Amphilophus astorquii* from crater Lake Apoyo.

There is a paucity of evidence about the genetic basis of early divergence between two lineages as they emerge from a common ancestor (reviewed in Coyne and Orr 2004; Nosil and Schluter 2011). For species that arose through allopatric speciation without exchanging genetic material, the random accumulation of genetic incompatibilities by time might explain their reproductive isolation. However, especially in stages of speciation with gene flow, it is of great interest to ask (1) whether few or many genes and where they are located, *e.g.*, clumped, in the genome; and (2) whether the same type of genes are involved in the process of speciation (Elmer and Meyer 2011; Feder *et al.* 2012). In only a few wild vertebrate populations have these questions been addressed via gene mapping and QTL mapping studies; thus, only very few crucial genes responsible for ecological divergence (*e.g.*, Colosimo *et al.* 2005; Chan *et al.* 2010) and speciation (Coyne and Orr 2004; Nosil and Schluter 2011) have been identified so far. The development of a Midas cichlid linkage map will aid substantially in identifying the genes that underlie phenotypically and ecologically important traits in fishes early during their adaptations to local conditions and the process of incipient speciation.

Researchers have long questioned why some cichlid fishes evolve in such a diverse and fast manner compared with other vertebrate lineages (Meyer 1990a; Meyer 1993; Wittbrodt *et al.* 1998), or even other fishes in the same environment (Elmer *et al.* in press). Behavioral and morphological characters that have been proposed to be responsible for the elevated speciation rate of cichlids include parental care, sexual selection, functional design, and phenotypic plasticity and polymorphisms (Meyer 1987; Stiassny and Meyer 1999; Salzburger 2009). A particular genomic architecture, perhaps derived from whole-genome duplications, has been proposed as a driver of rapid response to selection and higher diversification rates in fishes in general and perhaps cichlids in particular (Meyer and Schartl 1999; Kuraku and Meyer 2008). The evolution of a cichlid-specific physical order of particular genes along the chromosomes, and certain interactions between these genes, might have resulted in a genomic architecture in cichlid lineages that favored diversification (Salzburger 2009). As an alternative mechanism hypermutability of the genome or specific genomic regions might allow organisms to adapt at a higher pace to changing selection pressures. Through a greater neutral mutation rate, the chance that beneficial mutations might emerge is enhanced, therefore allowing neofunctionalization to occur faster, as has been proposed for the facilitation of morphological diversity in teleosts (Ravi and Venkatesh 2008).

The mechanisms responsible for the incomparable diversity of cichlids are unknown. Only an accumulation and comparison of more and more analyzed genomic resources, such as transcriptomes, whole genomes, and linkage maps of cichlids and related organisms can shed light on these unresolved questions. Some progress has already been made in this direction (Albertson *et al.* 2003; Lee *et al.* 2005; Sanetra *et al.* 2009; Elmer *et al.* 2010a; Fan *et al.* 2012; Cichlid Genome Consortium, 2006). The present study seeks to contribute to this much-needed resource and analysis with a Neotropical lineage and, based on the pedigree of parents to F_2_ offspring, give an estimate of a genome-wide mutation rate.

For decades, the analysis of genomic data were restricted to a few model organisms and organisms of economical importance. Next-generation sequencing platforms allow for the cost-effective sequencing of whole or partial genomes in a relatively short time; this is greatly accelerating genomic research in non-model organisms (Stapley *et al.* 2010; Elmer and Meyer 2011). Apart from whole genome sequencing, with restriction site-associated DNA sequencing (RADSeq) a powerful new tool to examine the genomes of organisms has become available (Baird *et al.* 2008; reviewed in Davey and Blaxter 2010). The advantage of RADSeq is the reduction of the genomic information of an organism to a usable and comparable fraction of homologous single nucleotide polymorphisms across a set of individuals. By choosing enzymes that differ in their cutting frequency, RADSeq allows for the development of variable numbers of repeatable genetic markers (RAD markers) to be sequenced by Illumina next-generation sequencing technology (Baird *et al.* 2008). RAD markers are polymorphic loci (*i.e.*, sequences with at least one single-nucleotide polymorphism [SNP]) within or between individuals. Here, we apply a newly developed double-digest RADSeq (ddRADSeq) protocol (based on Peterson *et al.* 2012) to create a linkage map of various species of Midas cichlids.

In the present study, we construct a linkage map with the F_2_ progeny of an interspecific cross between the benthic *A*. *astorquii* and the limnetic *A*. *zaliosus* Midas cichlids as a step toward uncovering the genetic basis of adaptive traits in this species complex. The linkage map will improve genomic resources for cichlid fishes in general, and Neotropical cichlids in particular, and along with whole-genome sequencing, contribute to insights into how cichlids achieved their astonishing phenotypic variability.

## Materials and Methods

### Pedigree

A wild-caught male *A. zaliosus* (2007, Lake Apoyo, Nicaragua) and a wild-caught female *A. astorquii* (2007, Lake Apoyo, Nicaragua) were reared separately in the laboratory until sexual maturity and then crossed to obtain F_1_ hybrid progeny. The offspring were raised to sexual maturity in a community tank to allow full-sib pairing. When a pair formed, they were moved to a separate tank and after spawning naturally their eggs were harvested and reared in another tank. F_2_ offspring were killed when they reached approximately 2 cm total length to get enough tissue for DNA extraction. The F_1_ pair that had the greatest number of F_2_ offspring was chosen for construction of the linkage map.

### ddRADSeq library preparation and sequencing

Genomic DNA was extracted from the two parents (“P generation”), the F_1_ hybrid pair and 347 F_2_ individuals using the QIAGEN DNeasy Blood & Tissue Kit following the manufacturer’s instructions including RNase A treatment. Then, 1 μg of DNA template from each individual was double-digested using the restriction enzymes *Pst*I-HF (20 units/reaction) and *Msp*I (20 units/reaction) in one combined reaction for 3 hr at 37°. Subsequently, each fragmented sample was purified using the QIAGEN MinElute Reaction Cleanup Kit, eluted in 20 μL of Elution Buffer (EB), and quantified. Fragments were then ligated to P1 adapters that bind to *Pst*I-HF created restriction sites and P2 adapters binding to overhangs generated by *Msp*I (see Supporting Information, Table S1 for adapter sequences). In each reaction, ∼0.4 μg of DNA, 1 μL of P1 adapter (10 μM), 1 μL of P2 adapter (10 μM), 1 μL of T4 ligase (1,000 U/μL), 4 μL of 10× T4 ligation buffer, and double-distilled water were combined to a total volume of 40 μL. The ligation was processed on a polymerase chain reaction (PCR) machine using the following conditions: 37° for 30 min, 65° for 10 min, followed by a decrease in temperature by 1.3° per minute until reaching 20°.

After we ligated each individual’s DNA to the adapters, samples were pooled and size-selected from an agarose gel. A preliminary Illumina sequencing run containing the parental and F_1_ DNA revealed that the pilot analysis gel size range of 250−500 bp resulted in sequencing too many fragments and therefore a range of ∼330−400 bp was chosen for subsequent libraries. Manual size selection from agarose gel electrophoresis was performed on the library containing the parental and F_1_ samples and samples were then cleaned using the QIAGEN MinElute Gel Purification Kit and eluted in 10 μL of EB. Fifty F_2_ individuals were pooled per library and size selected using Pippin Prep technology (Sage Science, Beverly, MA). Seven PCRs (several are necessary to minimize PCR bias) were performed in replicate on size-selected fragments [(Peterson *et al.* 2012) Figure 1]. Each PCR contained 10−20 ng of library DNA template, 4 μL of dNTPs (100 mM), 4.0 μL of 5× Phusion HF buffer (NEB), 0.2 μL of Phusion *Taq* polymerase (NEB), 1.0 μL of each RAD primer (10 μM), and filled up to 20 μL with double-distilled water. PCR conditions were performed as follows 98° (30 sec), [98° (10 sec), 65° (30 sec), 72° (30 sec)] × 10, 72° (300 sec). Gel electrophoresis was performed on pooled products to remove remaining oligo dimers and other contaminants. Fragments ranging from 330 to 400 bp were excised from a gel after electrophoresis and cleaned using the QIAGEN MinElute Gel Extraction Kit and eluted in 10 μL of EB. The eight libraries (one containing the parental and F_1_ DNA and the remainder seven each containing 50 F_2_ individuals) were diluted to 1 nM and sequenced single end to 150 bp length with the Illumina TruSeq SBS Kit v5 in eight lanes on an Illumina Genome Analyzer IIx (GeCKo: Genomics Center University of Konstanz) over two consecutive runs, each containing a single PhiX control lane.

**Figure 1:**
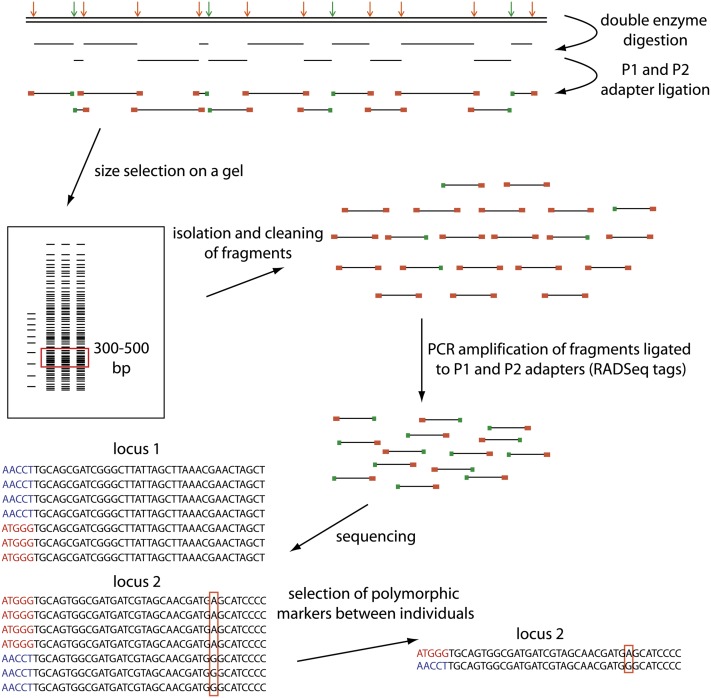
Schematic of the ddRADSeq protocol. Arrows indicate restriction sites for a rare-cutting enzyme in green and a frequent-cutting enzyme in orange. P1 adapters specifically bind to the rare-cutting enzyme (green) and P2 adapters bind to the frequent-cutter (orange) restriction sites. Each individual contains a different barcode of five nucleotide bases specified by the P1 adapter. Sequenced RAD tags start with the barcode (individual one: red barcode, individual two: blue barcode). The bioinformatic software Stacks allows for the detection of single nucleotide polymorphisms between individuals (outlined by red box in sequence alignment).

### Linkage mapping analysis

Bioinformatic processing of the reads (*i.e.*, RADSeq tags) was performed using Stacks (Catchen *et al.* 2011). Reads were trimmed to a length of 110 bp, cleaned from erroneous and low-quality reads, and sorted by individual. Processing of the 347 F_2_-offspring individuals was performed using the denvo_map.pl script, which creates stacks of loci (*i.e.*, RADSeq stacks) within individuals, followed by building a catalog of loci present in the two parents and then mapping all stacks from the offspring to the parental catalog and finally creating the offspring genotypes for those loci. Multiple SNPs in one RADSeq stack were treated as a single polymorphic marker (*i.e.*, one RAD marker). The parameters were optimized and set as follows: the minimum amount of identical reads to create a stack in each parent and progeny was set to three (−m 3, −P 3) the number of mismatches allowed between loci from the parents was set to three (−n 3), and highly repetitive RAD tags were removed or disjointed (−t), other parameters were set to default.

JoinMap 4 (Van Ooijen 2006) was used to perform linkage-mapping analysis. Genotypes were exported from Stacks and were filtered to only include those markers for which there were genotype calls in at least 90 F_2_ individuals with a coverage of at least 5×. Linkage analyses were performed using all markers (of the type aa/bb, aa/ab, ab/aa, ab/ab, ab/ac, ab/cc in the P generation as defined by Stacks) containing alleles from the F_1_ pair that could be matched with confidence to each parental unit and did not deviate from Mendelian segregation ratios. Markers under segregation distortion (*P* = 0.05−0.001) were added step by step from the less significant to the most and their effect on the map was checked. Marker groupings were calculated using an independent logarithm of odds (LOD) score of 6.0, but the number of calculated linkage groups did not change between LOD scores of 6.0 to a more stringent LOD score of 10.0. Each linkage group was calculated using the regression mapping algorithm and Kosambi’s mapping function (Kosambi 1943) with the following thresholds: recombination frequency of 0.400, LOD value of 1.0, and a goodness-of-fit jump of 5.00. Marker order was optimized by performing a ripple function after each added locus.

### Comparative analysis

All RAD markers used in the linkage map were indexed with the respective linkage group and position and compared with the genomes of the fishes tilapia (*Oreochromis niloticus*; version from 25.01.2012), stickleback (*Gasterosteus aculeatus*; version BROADS1.61), and medaka (*Oryzias latipes*; version MEDAKA1.48) using blastn searches and an e-value cut-off of 10^−15^ for tilapia (a basal cichlid) and 10^−10^ for stickleback and medaka. Tilapia was used as a representative of the African cichlid lineage, whereas stickleback and medaka were used as phylogenetically close teleost outgroups to the cichlids. If a marker mapped to more than one locus it was either excluded or, if the difference in the bit score between best hit and second-best hit was more than 20, the best hit was retained. Markers that mapped to unlinked scaffolds were excluded from the synteny analyses. Synteny was calculated in percentage of markers mapping to a single orthologous linkage group in contrast to those mapping to different linkage groups. Markers that solely map to a single linkage group, *i.e.*, if no other markers mapped to that same linkage group, were excluded.

### Mutation rate estimation

*De novo* mutations are expected to be recognized as high-confidence SNPs present in one or a few offspring but not in the parents. Loci from two F_1_ individuals and eight randomly chosen F_2_ individuals with high coverage were compared to the parental (P) loci and checked for SNPs only present in the progeny. The coverage for a locus to be included in the analysis was set to at least 8× (minimally 4× per allele to reduce the chance of candidate new mutations being sequencing errors). In addition, an empirical distribution of allele-to-allele coverage ratio was estimated from all heterozygous RAD markers in the F_2_ generation. New mutation candidates in the progeny were checked against this distribution, and only those mutations that had an allele ratio within the 95% confidence interval of the loci present in the empirically estimated distribution were retained. This step yielded loci with a ratio lower than 0.5 or greater than 2.0 to be discarded and was performed to exclude candidate loci with odd allele-to-allele ratios (*e.g.*, repetitive sequences confounded with alleles). For example, a locus with a mutation candidate and 10 reads in total, including seven reads of the original allele and three reads of the allele with the new mutation, would be excluded because the allele-to-allele ratio is lower than 0.5.

## Results

### SNP and marker detection

**Figure 2:**
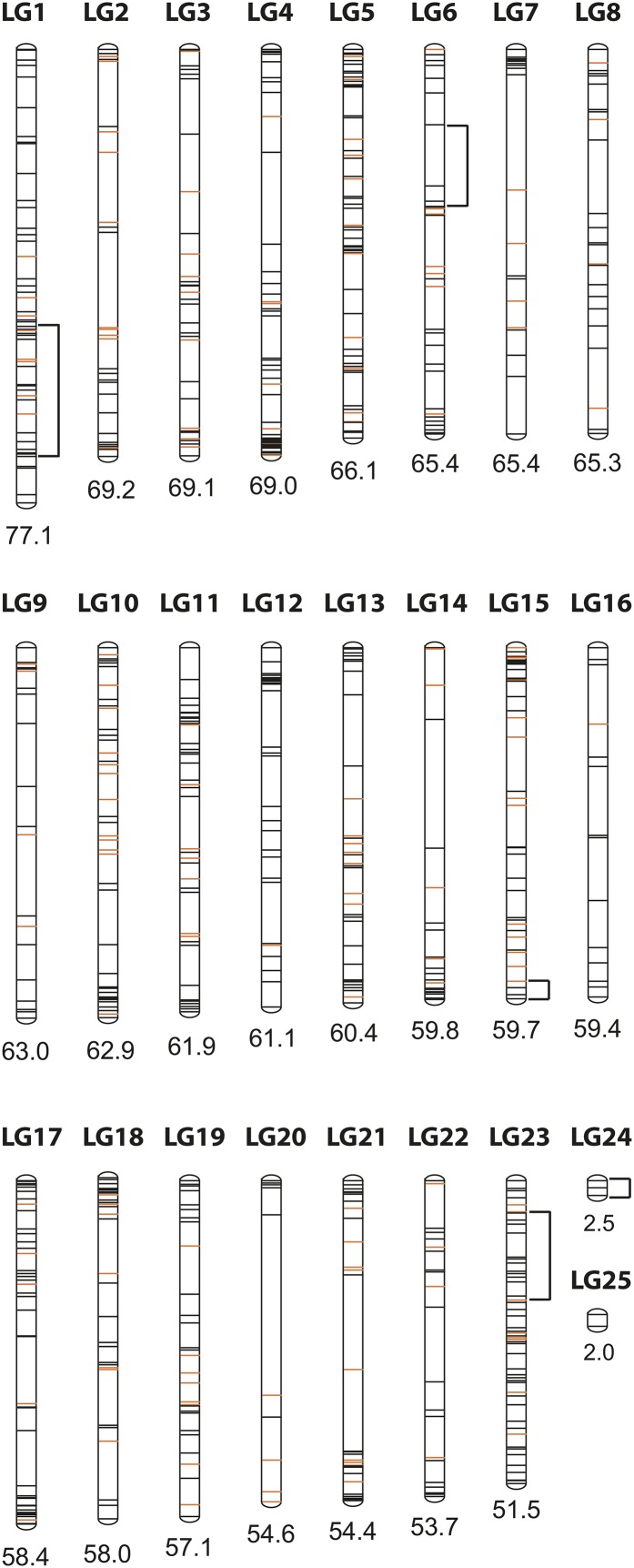
Linkage groups constructed with 755 RAD markers and ordered by size. Blocks of segregation distortion are indicated by brackets. Markers used for synteny analyses are shown in orange. For further information on marker IDs, position, segregation distortion and markers used in synteny analyses see, Table S3.

A total of 254 million reads were used, with an average of 31.8 million reads per library (SD: 3.1 million reads). On average, approximately 588,000 reads were obtained per individual (SD: 201,454 reads) with 15× coverage per read (SD: 5.1×). Within the parental *A*. *astorquii* individual, a SNP frequency of 0.80 SNPs/kb (11,172 SNPs in 139,109 loci) and for *A*. *zaliosus* 0.89 SNPs/kb (12,764 SNPs in 142,740 loci) were detected (Table S2). Of 118,213 loci that were scored in both parents, 12,429 were polymorphic with one to three SNPs, with a total of 15,035 SNPs and a frequency of 1.2 SNPs/kb. Of those loci that were polymorphic, 18.5% were fixed between parents. A total of 38,203 loci were detected in the parents and were present in at least 90 of the 343 F_2_ individuals. The number of loci shared between parents and F_2_ progeny was smaller than that between parents and F_1_ because the F_2_ progeny gel size range was narrower and therefore included fewer fragments. A total of 3,184 loci that were shared between parents, F_1_ and F_2_s were polymorphic and retained for further analyses. Of these loci, 755 were informative (alleles that segregated in a 1:2:1 segregation ratio in the F_2_ generation) and could serve for the construction of the linkage map (Figure 2). Because different libraries did not exactly contain the same loci and some of the loci did not pass the coverage threshold, we obtained ∼25% missing data. Almost 50% of the loci were present in more than 300 individuals (Figure S1).

### Linkage groups

Of the 347 F_2_ individuals genotyped with ddRADSeq, four were excluded because of low coverage (<6× per individual; see Table S3 for genotype data). From linkage mapping analysis, 25 linkage groups were identified with a total size of 1427 cM and an average marker spacing distance of 1.95 cM (Figure 2, Table S4). Linkage groups were between 51.5 and 77.1 cM in length except for the two small linkage groups LG24 and LG25 with 2.5 and 2.0 cM, respectively. With the exception of two small linkage groups with only two and three linked markers, all linkage groups consisted of at least 10 markers (minimum: 10 markers, maximum: 54 markers). On average, a typical linkage group comprised 30 markers (Table S4). A total of 17 markers (2.3% of all markers) were completely linked (no recombination was observed between them). The maximum distance between two markers on a single linkage group was 30.7 cM on LG20.

### Segregation distortion

Almost 10% of all markers (74 of 755 markers) deviated from Mendelian segregation ratios with a *P*-value between 0.05 and 0.001 (Table S3). Markers that were distorted significantly with *P* < 0.0001 were not considered because ratios that are too skewed might create inaccurate distances and false linkages (Cervera *et al.* 2001). Closely linked markers with similar patterns of segregation distortion probably can be attributed to biological causes, rather than genotyping error. Of these 74 markers, in fact 19 could be found on LG1 (39% distorted markers on LG1) with most of them directly strung together. In all these markers the ratio was skewed toward a higher frequency of the paternal allele. Apart from the large segregation distortion block on LG1, four other smaller blocks of segregation distortion were identified: five adjacent markers on LG6, three markers in close proximity at the distal part of LG15, seven markers on LG23 spanning a distance of ∼15 cM and all three markers on LG24. LG6 and LG24 distorted markers exhibit a higher ratio of the maternally inherited allele, whereas distorted markers both on LG7 and LG23 display an excess of heterozygotes. Thus, half of all distorted markers (37 of 74) are placed on these five blocks (Figure 2, Table S3).

### Comparative genomics

**Figure 3:**
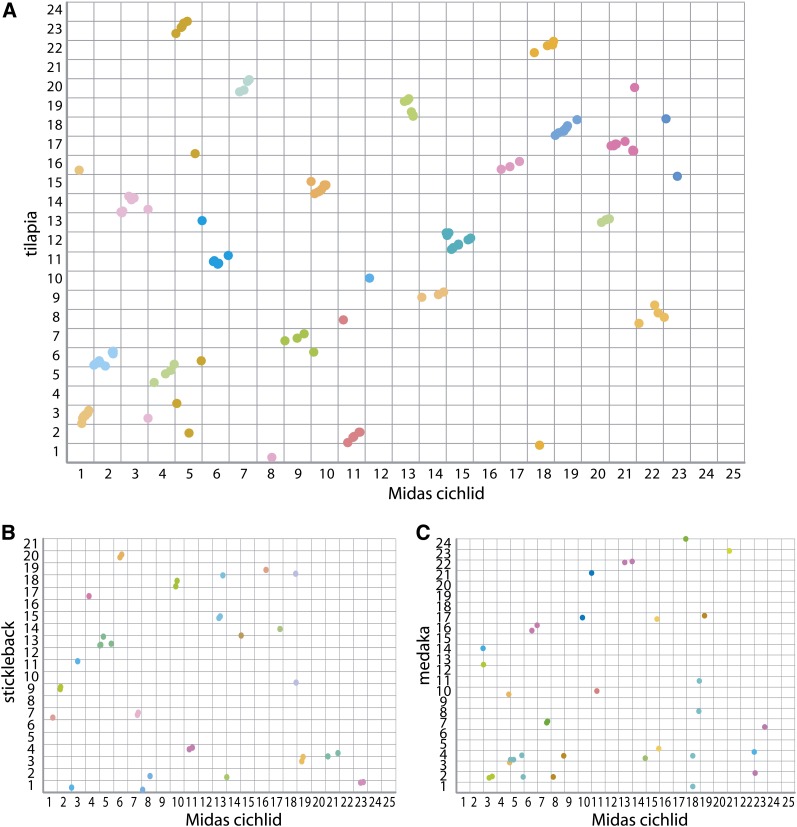
Oxford grid showing relationship between Midas cichlid and other teleost fishes. Each linkage group (in the case of medaka each chromosome) is size-normalized to equal one and marker positions were placed relative to their respective linkage group (or chromosome) size. Oxford grids showing marker relationships between (A) tilapia and Midas cichlid, (B) stickleback and Midas cichlid, and (C) medaka and Midas cichlid. Color refers to markers found on a Midas linkage group.

The vast majority of markers present in any given particular Midas cichlid linkage group also map to a single orthologous linkage group in tilapia (92 of 107 markers that uniquely mapped to the tilapia genome, excluding those markers that mapped to a single linkage group with only this one syntenic marker), a basal African cichlid that last shared a common ancestor at least 60−90 million years ago (Salzburger and Meyer 2004). Four linkage groups in tilapia (LGs 1, 4, 10, and 21) and six linkage groups in Midas (LGs 8, 12, 16, 23, 24, and 25) are not represented by more than two markers that map to any orthologous linkage group. Midas cichlid linkage group 23 mapped to 14 loci of different scaffolds in the tilapia genome. For the other five Midas linkage groups that did not map to orthologous linkage groups in tilapia, this is caused by an insufficient number of markers used to anchor the tilapia genome (Table S5) or small linkage group sizes (due to insufficient data in tilapia genomic resources) in tilapia. In addition to the linkage groups that share homologous markers (conserved synteny), the respective position of these markers (linkage order) is mostly the same, suggesting retained syntenic relationships among those loci (Figure 3). However, divergent marker orders, presumably caused by intrachromosomal rearrangements, can be detected to some extent between the Midas cichlid and tilapia (*e.g.*, LGs 13, 15, and 21 in the Midas cichlid). In general, however, it is clear that overall levels of synteny between these two cichlid lineages are quite highly conserved, with 86% of all markers mapping to a single orthologous linkage group when those linkage groups containing two or more markers are considered.

As expected, fewer homologous markers were found for stickleback (44 markers) and medaka (38 markers) compared with tilapia and the Midas cichlid (Figure 3). The stickleback shows many markers of conserved synteny (78%) with the Midas cichlid. In contrast, fewer markers show conserved synteny (38%) between medaka and Midas cichlids.

### Mutation rate

Two new mutations in the F_1_ progeny and five new mutations in the F_2_ progeny were found. The same mutation was observed in two different F_2_ individuals and was therefore only counted as one mutation. The F_1_ mutation rate was calculated to 7.4 × 10^−8^ and the F_2_ 5.8 × 10^−8^, leading to an average mutation rate of 6.6 × 10^−8^ mutations per nucleotide per generation.

## Discussion

Here, we report the first linkage map of a Neotropical cichlid species. It will serve as important addition to the genomic resources for cichlid fishes and facilitate in searching for genes that contribute to ecological and phenotypic diversity in Midas cichlids in particular and cichlids more generally. With a total of 25 linkage groups, the present linkage map closely matches the number of chromosomes (n = 24) in the Midas cichlid (Feldberg *et al.* 2003). The small groups LG24 and LG25 might represent larger linkage groups or collapse with another linkage group when further markers can be incorporated. Alternatively, in the case of LG24, markers in greater distance might not have been identified because of highly distorted segregation ratios rather than missing genotypes. Significant parts of a linkage group might be completely missing if it contains distorted regions (Cervera *et al.* 2001; Doucleff *et al.* 2004). Alternatively, the distorted markers constitute false linkages (*e.g.*, Nelson *et al.* 2010), though at least in the case of LG24 this seems improbable given that all three markers are in very close proximity and a small number of recombination events have occurred between them.

Several genes associated with phenotypic divergence between cichlid species have been identified to date (Kijimoto *et al.* 2005; Kobayashi *et al.* 2006; Albertson and Kocher 2006; Salzburger *et al.* 2007; Roberts *et al.* 2009; Fan *et al.* 2011; Roberts *et al.* 2011). In African cichlids, a specific linkage group (LG5) has been proposed to constitute a “speciation chromosome” containing genes responsible for sex determination, tooth shape, visual sensitivity, and color pattern (Streelman and Albertson 2006). This linkage group is orthologous to the Midas LG4. It will be interesting to test whether these genes and this particular linkage group are also important for traits under selection in Neotropical cichlids.

As the number of genetic markers that can be quickly generated has exploded with new sequencing technology, the limiting factor for constructing a high-resolution linkage map is the biological resources: the number of individuals and recombination events. Thousands of markers can be produced in a short time with ddRADSeq but high-resolution mapping can only be achieved by maximizing the number of recombinations, *i.e.*, the individuals. Under laboratory conditions, Midas cichlids can spawn naturally almost once per month with more than a 300 offspring per clutch, which allows for harvesting a high number of individuals. Because of the Midas cichlids’ amenable life history, we were able to create a genetic map based on more than 340 F_2_ individuals from one F_1_ family. The number of markers that can be generated and the amount of missing data that is accepted constitute a trade-off. When minimizing missing data, markers that are not present in all individuals (*e.g.*, through library effects or coverage thresholds) will be lost. Accordingly, while maximizing the number of loci, more missing data are accepted, which might result in incorrect distances in linkage map construction and in QTL analyses (Hackett and Broadfoot 2003).

To perform synteny analyses and achieve a high marker density, we allowed more missing data for the purpose of this study. Compared with the five other linkage maps that have been constructed with RADSeq technology so far (Amores *et al.* 2011; Baxter *et al.* 2011; Chutimanitsakun *et al.* 2011; Pfender *et al.* 2011; Parnell *et al.* 2012), the map of the present study has the second highest number of progeny genotyped for markers and hence a high number of recombinations. The gar linkage map consisted of more markers (∼5500), although it was based on fewer individuals (Amores *et al.* 2011). The average spanning distance between two markers is 1.95 cM in our study, which is the lowest value published to date for a RADSeq study. In comparison with the published cichlid linkage maps from *A. burtoni* (Sanetra *et al.* 2009), tilapia (Lee *et al.* 2005), and Lake Malawi cichlids (Albertson *et al.* 2003; Parnell *et al.* 2012), the present Midas cichlid linkage map has the second highest number of individuals, the highest number of markers, and lowest average distance between two markers. This result highlights the promise of next-generation sequencing based genotyping, and especially the ddRADSeq protocol, for linkage mapping experiments when biological material is sufficient.

Although *A*. *astorquii* and *A*. *zaliosus* have clearly distinct ecologies, their divergence time is very recent [should be less than 10,000 years (Barluenga *et al.* 2006) and maximally 20,000 years (Kutterolf *et al.* 2007)]. For this reason, only ∼10.5% of our markers were polymorphic and of those 18.5% were fixed between species. Given this young divergence, it is of great interest how these species developed and maintain reproductive isolation. Besides premating isolation, chromosomal rearrangements or genetic incompatibilities might act as isolating barriers to gene flow in postzygotic reproduction (reviewed in Coyne and Orr 2004). As the species are young and can be crossed easily, incompatible loci seems more probable than large genomic rearrangements (Moyle and Graham 2005). Genetic incompatibilities between species detected on the basis of segregation distortion have been suggested in many cases, including lake whitefish (Rogers and Bernatchez 2007; Rogers *et al.* 2007), *Drosophila* (Phadnis and Orr 2009), *Nasonia* (Gadau *et al.* 1999; Niehuis *et al.* 2008), and some plant species (Foolad *et al.* 1995; Whitkus 1998; Fishman *et al.* 2001; Myburg *et al.* 2004).

In our linkage mapping analysis, we found that 10% of the markers deviated (*P* < 0.05) from expected Mendelian segregation ratio. Single markers deviating from the expected ratio might result from genotyping errors, though blocks of deviating linked markers as on LGs1, 6, 15, 23, and 24 probably have a biological basis and might be caused by segregation distortion genes (*e.g.*, Lyttle 1993) or genetic incompatibilities by differential fixation of alleles between parents (Dobzhansky 1937; Muller 1942; Fishman and Willis 2001). If genomic incompatibilities between species exist, chromosomal regions should consistently exhibit segregation distortion. As our data only originates from a single family, further testing in laboratory bred or wild *A. astorquii* and *A. zaliosus* hybrids will be necessary (Danzmann and Gharbi 2001) to reveal whether the five linkage groups exhibiting blocks of distorted markers result from interspecific genetic incompatibilities.

Genomic architecture has been suspected of being a main factor contributing to the cichlids' evolutionary success in terms of biodiversity (Salzburger 2009). The comparative genomic analysis between the Midas cichlid and the tilapia reveals a highly conserved synteny with 86% of the markers mapping to a single orthologous linkage group. This finding points toward a high level of conserved synteny between Neotropical and African cichlids, in spite of their relatively ancient split (60 mya according to Vences *et al.* 2001; Matschiner *et al.* 2011; but see Farias *et al.* 1999; Salzburger and Meyer 2004; Azuma *et al.* 2008). A relatively high level of conservatism in number of chromosomes across cichlid genera and species is observed (see Feldberg *et al.* 2003), indicating that interchromosomal rearrangements are rare. This observation and our results suggest that at least large-scale chromosomal rearrangements are probably not a main factor contributing to the species richness in cichlids and also agree with previous studies demonstrating a high degree of conserved synteny between cichlid genomes (Sanetra *et al.* 2009). As a comparison, the human and lemur lineages diverged ∼74 mya (*e.g.*, Huchon *et al.* 2007), although their number of chromosomes differs considerably (*Homo sapiens*: 23 chromosomes; *Microcebus murinus*: 33 chromosomes), including several interchromosomal rearrangements that occurred after their split (Dutrillaux 1979).

Medaka and stickleback show less conserved synteny with the Midas cichlid (medaka: 38%, stickleback: 78% markers with conserved synteny). Although the data for genomic comparisons between the Midas cichlid and the noncichlid fish species stickleback and medaka are limited because of the large phylogenetic distance of more than 100 million years since these lineages last shared a common ancestor (Matschiner *et al.* 2011), it allows for two conclusions: (1) the degree of conserved synteny with these species is lower than compared with tilapia and (2) medaka shares considerably less conserved synteny with the Midas cichlid compared with stickleback. As chromosomal rearrangements leading to linkage and synteny disruptions accumulate with time, stickleback (divergence time ∼140 mya, Matschiner *et al.* 2011) and medaka (divergence time ∼100 mya, Matschiner *et al.* 2011) display less conserved synteny to the Midas cichlid, as expected, compared with the younger split of African *vs.* Neotropical cichlids. However, synteny disruption seems to be greater between medaka and the Midas cichlid compared with stickleback, which was unexpected because most phylogenies place the stickleback basal to cichlids and medaka (Chen *et al.* 2003; Mabuchi *et al.* 2007; Azuma *et al.* 2008, Matschiner *et al.* 2011). Incorrect homology determination or mapping errors might account for a lower degree of conserved synteny between the Midas cichlid and medaka. Alternatively, the medaka lineage has undergone considerable more rearrangements compared with cichlids and stickleback, and/or phylogenetic relationships are incorrect, as relationships between these teleosts remain controversial (Chen *et al.* 2003; Steinke *et al.* 2006).

The mutation rate (*u*) estimation of 6.6 × 10^−8^ per site per generation in Midas cichlids indicates a somewhat elevated mutation rate compared to estimates from other vertebrates [mouse: 3.8 × 10^−8^, human: 3.0 × 10^−8^ (Lynch 2010)]. A mutation rate based on the synonymous substitution rate and UTRs from pooled Midas cichlid expressed sequence tags yielded an estimate (∼1.25 × 10^−6^ per site per year) higher than that of the present study, although it was thought to be elevated by population-level polymorphism (Elmer *et al.* 2010a). For mitochondrial DNA control region, the substitution rate has been estimated as 7.1 × 10^−8^ per site per year (Barluenga and Meyer 2004), which, despite the fact that control region should have a high mutation rate, is fairly well in agreement with our current genome-wide mutation rate estimate. However, all calculations of mutation or substitution in recently diverged populations differ because of the role of lineage sorting in decreasing rates with time (*i.e.*, pedigree *vs.* population *vs.* phylogenetic rates) [see *e.g.*, (Ho *et al.* 2005; Subramanian *et al.* 2009)]. Although vertebrate estimates only include model species such as human, mouse, and rat, this finding is congruent with interspecific comparisons of mutation rates in teleost compared with mammalian lineages that revealed that teleosts exhibit a higher neutral mutation rate (Ravi and Venkatesh 2008). It might be argued that an elevated mutation rate might explain the teleost morphological diversity and species richness, since the chance that a beneficial mutation arises on which selection can act is increased. More estimates of vertebrate mutation rates are needed in order to ascertain whether the cichlid lineage, and species-rich lineages in general, exhibit higher mutation rates.

Cichlid fishes represent wonderful examples for phenotypic diversity and convergence, rapid speciation, and adaptive radiation (Fan *et al.* 2012). Yet, we have still to understand how the underlying genetics of this diversity operate to translate into the ubiquitous phenotypic richness of this evolutionary lineage. The present linkage map adds valuable genomic information to tackle these fundamental questions in evolutionary biology. Future genome-wide analyses on the level of populations, species, and among distantly related lineages in cichlid fishes will profit from this resource and it will help to shed light on the genomics of adaptation in this extraordinarily diverse evolutionary lineage.

## Supplementary Material

Supporting Information

## References

[bib1] Albertson R. C. Kocher T. D., 2006 Genetic and developmental basis of cichlid trophic diversity. Heredity 97: 211–221 16835594 10.1038/sj.hdy.6800864

[bib2] Albertson R. C. Streelman J. T. Kocher T. D., 2003 Directional selection has shaped the oral jaws of Lake Malawi cichlid fishes. Proc. Natl. Acad. Sci. USA 100: 5252–5257 12704237 10.1073/pnas.0930235100 PMC154331

[bib3] Amores A. Catchen J. Ferrara A. Fontenot Q. Postlethwait J. H., 2011 Genome evolution and meiotic maps by massively parallel DNA sequencing: spotted gar, an outgroup for the teleost genome duplication. Genetics 188: 799–808 21828280 10.1534/genetics.111.127324 PMC3176089

[bib4] Azuma Y. Kumazawa Y. Miya M. Mabuchi K. Nishida M., 2008 Mitogenomic evaluation of the historical biogeography of cichlids toward reliable dating of teleostean divergences. BMC Evol. Biol. 8: 215 18651942 10.1186/1471-2148-8-215 PMC2496912

[bib5] Baird N. A. Etter P. D. Atwood T. S. Currey M. C. Shiver A. L., 2008 Rapid SNP discovery and genetic mapping using sequenced RAD markers. PLoS ONE 3: e3376 18852878 10.1371/journal.pone.0003376 PMC2557064

[bib95] Barlow G. W. Munsey J. W., 1976 The red devil–Midas-arrow cichlid species complex in Nicaragua, pp. 359–369 Investigations of the Ichthyology of Nicaraguan Lakes, edited by Thorson T. B. University of Nebraska Press, Lincoln, NE

[bib6] Barluenga M. Meyer A., 2004 The Midas cichlid species complex: incipient sympatric speciation in Nicaraguan cichlid fishes? Mol. Ecol. 13: 2061–2076 15189226 10.1111/j.1365-294X.2004.02211.x

[bib7] Barluenga M. Meyer A., 2010 Phylogeography, colonization and population history of the Midas cichlid species complex (*Amphilophus* spp.) in the Nicaraguan crater lakes. BMC Evol. Biol. 10: 326 20977752 10.1186/1471-2148-10-326 PMC3087546

[bib8] Barluenga M. Stölting K. N. Salzburger W. Muschick M. Meyer A., 2006 Sympatric speciation in Nicaraguan crater lake cichlid fish. Nature 439: 719–723 16467837 10.1038/nature04325

[bib9] Baxter S. W. Davey J. W. Johnston J. S. Shelton A. M. Heckel D. G., 2011 Linkage mapping and comparative genomics using next-generation RAD sequencing of a non-model organism. PLoS ONE 6: e19315 21541297 10.1371/journal.pone.0019315 PMC3082572

[bib10] Baylis J. R., 1976 a A quantitative study of long-term courtship: I. Ethological isolation between sympatric populations of the Midas cichlid, *Cichlasoma citrinellum*, and the arrow cichlid. Behaviour 59: 59–69

[bib11] Baylis J. R., 1976 b A quantitative study of long-term courtship: II. A comparative study of the dynamics of courtship in two New World cichlid fishes. Behaviour 59: 117–140

[bib12] Berra T., 2007 Freshwater Fish Distribution, University of Chicago Press, Chicago

[bib13] Catchen J. M. Amores A. Hohenlohe P. Cresko W. Postlethwait J. H., 2011 Stacks: building and genotyping loci de novo from short-read sequences. G3 (Bethesda) 1: 171–182 22384329 10.1534/g3.111.000240 PMC3276136

[bib14] Cervera M. T. Storme V. Ivens B. Gusmão J. Liu B. H., 2001 Dense genetic linkage maps of three Populus species (*Populus deltoides*, *P. nigra* and *P. trichocarpa*) based on AFLP and microsatellite markers. Genetics 158: 787–809 11404342 10.1093/genetics/158.2.787 PMC1461694

[bib15] Chan Y. F. Marks M. E. Jones F. C. Villarreal G. Shapiro M. D., 2010 Adaptive evolution of pelvic reduction in sticklebacks by recurrent deletion of a Pitx1 enhancer. Science 327: 302–305 20007865 10.1126/science.1182213 PMC3109066

[bib16] Chen W. J. Bonillo C. Lecointre G., 2003 Repeatability of clades as a criterion of reliability: a case study for molecular phylogeny of Acanthomorpha (Teleostei) with larger number of taxa. Mol. Phylogenet. Evol. 26: 262–288 12565036 10.1016/s1055-7903(02)00371-8

[bib17] Chutimanitsakun Y. Nipper R. W. Cuesta-Marcos A. Cistue L. Corey A., 2011 Construction and application for QTL analysis of a Restriction Site Associated DNA (RAD) linkage map in barley. BMC Genomics 12: 4 21205322 10.1186/1471-2164-12-4 PMC3023751

[bib18] Cichlid Genome Consortium, 2006 Available at: http://www.cichlidgenome.org. Accessed: November 28, 2012

[bib19] Colosimo P. F. Hosemann K. E. Balabhadra S. Villarreal G. Dickson M., 2005 Widespread parallel evolution in sticklebacks by repeated fixation of ectodysplasin alleles. Science 307: 1928–1933 15790847 10.1126/science.1107239

[bib20] Coyne J. A. Orr H. A., 2004 Speciation. Sinauer Associates, Sunderland, MA

[bib21] Danzmann R. Gharbi K., 2001 Gene mapping in fishes: a means to an end. Genetica 111: 3–23 11841175 10.1023/a:1013713431255

[bib22] Davey J. W. Blaxter M. L., 2010 RADSeq: next-generation population genetics. Brief. Funct. Genomics 9: 416–423 21266344 10.1093/bfgp/elq031 PMC3080771

[bib23] Dobzhansky T., 1937 Genetics and the Origin of Species. Columbia University Press, New York

[bib24] Doucleff M. Jin Y. Gao F. Riaz S. Krivanek A. F., 2004 A genetic linkage map of grape, utilizing *Vitis rupestris* and *Vitis arizonica*. Theor. Appl. Genet. 109: 1178–1187 15292989 10.1007/s00122-004-1728-3

[bib25] Dutrillaux B., 1979 Chromosomal evolution in primates: Tentative phylogeny from *Microcebus murinus* (Prosimian) to man. Hum. Genet. 48: 251–314 112030 10.1007/BF00272830

[bib26] Elmer K. R. Meyer A., 2011 Adaptation in the age of ecological genomics: insights from parallelism and convergence. Trends Ecol. Evol. 26: 298–306 21459472 10.1016/j.tree.2011.02.008

[bib27] Elmer K. R. Fan S. Gunther H. M. Jones J. C. Boekhoff S., 2010 a Rapid evolution and selection inferred from the transcriptomes of sympatric crater lake cichlid fishes. Mol. Ecol. 19: 197–211 20331780 10.1111/j.1365-294X.2009.04488.x

[bib28] Elmer K. R. Kusche H. Lehtonen T. K. Meyer A., 2010 b Local variation and parallel evolution: morphological and genetic diversity across a species complex of neotropical crater lake cichlid fishes. Philos. Trans. R. Soc. B Biol. Sci. 365: 1763–1782 10.1098/rstb.2009.0271 PMC2871887 20439280

[bib29] Elmer K. R. Lehtonen T. K. Kautt A. Harrod C. Meyer A., 2010 c Rapid sympatric ecological differentiation of crater lake cichlid fishes within historic times. BMC Biol. 8: 60 20459869 10.1186/1741-7007-8-60 PMC2880021

[bib30] Elmer K. R. Lehtonen T. K. Fan S. Meyer A., 2013 Crater lake colonization by Neotropical cichlid fishes. Evolution (in press) DOI: 10.111/j.1558–5646.2012.01755.x 10.1111/j.1558-5646.2012.01755.x 23289578

[bib31] Fan S. Elmer K. R. Meyer A., 2011 Positive Darwinian selection drives the evolution of the morphology-related gene, EPCAM, in particularly species-rich lineages of African cichlid fishes. J. Mol. Evol. 73: 1–9 21811860 10.1007/s00239-011-9452-5

[bib32] Fan S. Elmer K. R. Meyer A., 2012 Genomics of adaptation and speciation in cichlid fishes: recent advances and analyses in African and Neotropical lineages. Philos. Trans. R. Soc. B Biol. Sci. 367: 385–394 10.1098/rstb.2011.0247 PMC3233715 22201168

[bib33] Farias I. P. Ortí G. Sampaio I. Schneider H. Meyer A., 1999 Mitochondrial DNA phylogeny of the family Cichlidae: monophyly and fast molecular evolution of the Neotropical assemblage. J. Mol. Evol. 48: 703–711 10229574 10.1007/pl00006514

[bib35] Feder J. L. Egan S. P. Nosil P., 2012 The genomics of speciation-with-gene-flow. Trends Genet. 28: 342–350 22520730 10.1016/j.tig.2012.03.009

[bib36] Feldberg E. Porto J. I. R. Bertollo L. A. C., 2003 Chromosomal changes and adaptation of cichlid fishes during evolution, pp. 285–308 Fish Adaptation, edited by Val A. L. Kapoor B. G. Science Publishers Inc, Enfield, NH

[bib37] Fishman L. Willis J. H., 2001 Evidence for Dobzhansky-Muller incompatibilities contributing to the sterility of hybrids between *Mimulus guttatus* and *M. nasutus*. Evolution 55: 1932–1942 11761055 10.1111/j.0014-3820.2001.tb01311.x

[bib38] Fishman L. Kelly A. J. Morgan E. Willis J. H., 2001 A genetic map in the *Mimulus guttatus* species complex reveals transmission ratio distortion due to heterospecific interactions. Genetics 159: 1701–1716 11779808 10.1093/genetics/159.4.1701 PMC1461909

[bib39] Foolad M. Arulsekar S. Becerra V. Bliss F., 1995 A genetic map of *Prunus* based on an interspecific cross between peach and almond. Theor. Appl. Genet. 91: 262–269 24169773 10.1007/BF00220887

[bib40] Fryer G. Iles T. D., 1972 The Cichlid Fishes of the Great Lakes of Africa: Their Biology and Evolution. Oliver and Boyd, Edinburgh

[bib41] Gadau J. Page R. E. Werren J. H., 1999 Mapping of hybrid incompatibility loci in Nasonia. Genetics 153: 1731–1741 10581280 10.1093/genetics/153.4.1731 PMC1460847

[bib42] Hackett C. Broadfoot L., 2003 Effects of genotyping errors, missing values and segregation distortion in molecular marker data on the construction of linkage maps. Heredity 90: 33–38 12522423 10.1038/sj.hdy.6800173

[bib43] Ho S. Y. W. Phillips M. J. Cooper A. Drummond A. J., 2005 Time dependency of molecular rate estimates and systematic overestimation of recent divergence times. Mol. Biol. Evol. 22: 1561–1568 15814826 10.1093/molbev/msi145

[bib44] Huchon D. Chevret P. Jordan U. Kilpatrick C. W. Ranwez V., 2007 Multiple molecular evidences for a living mammalian fossil. Proc. Natl. Acad. Sci. USA 104: 7495–7499 17452635 10.1073/pnas.0701289104 PMC1863447

[bib45] Kautt A. Elmer K. R. Meyer A., 2012 Genomic signatures of divergent selection and speciation patterns in a “natural experiment”, the young parallel radiations of Nicaraguan crater lake cichlid fishes. Mol. Ecol. 21: 4770–4786 22934802 10.1111/j.1365-294X.2012.05738.x

[bib46] Kijimoto T. Watanabe M. Fujimura K. Nakazawa M. Murakami Y., 2005 cimp1, a novel astacin family metalloproteinase gene from East African cichlids, is differentially expressed between species during growth. Mol. Biol. Evol. 22: 1649–1660 15858202 10.1093/molbev/msi159

[bib47] Kobayashi N. Watanabe M. Kijimoto T. Fujimura K. Nakazawa M., 2006 magp4 gene may contribute to the diversification of cichlid morphs and their speciation. Gene 373: 126–133 16517097 10.1016/j.gene.2006.01.016

[bib48] Kosambi D. D., 1943 The estimation of map distances from recombination values. Ann. Hum. Genet. 12: 172–175

[bib49] Kuraku S. Meyer A., 2008 Genomic analysis of cichlid fish ‘natural mutants’. Curr. Opin. Genet. Dev. 18: 551–558 19095433 10.1016/j.gde.2008.11.002

[bib50] Kutterolf S. Freundt A. Pérez W. Wehrmann H. Schmincke H.-U., 2007 Late Pleistocene to Holocene temporal succession and magnitudes of highly-explosive volcanic eruptions in west-central Nicaragua. J. Volcanol. Geotherm. Res. 163: 55–82

[bib51] Lee B. Y. Lee W. J. Streelman J. T. Carleton K. L. Howe A. E., 2005 A second-generation genetic linkage map of tilapia (*Oreochromis* spp.). Genetics 170: 237–244 15716505 10.1534/genetics.104.035022 PMC1449707

[bib52] Lehtonen T. K. Wong B. B. M. Lindström K. Meyer A., 2011 Species divergence and seasonal succession in rates of mate desertion in closely related Neotropical cichlid fishes. Behav. Ecol. Sociobiol. 65: 607–612

[bib53] Lynch M., 2010 Evolution of the mutation rate. Trends Genet. 26: 345–352 20594608 10.1016/j.tig.2010.05.003 PMC2910838

[bib54] Lyttle T. W., 1993 Cheaters sometimes prosper: distortion of Mendelian segregation by meiotic drive. Trends Genet. 9: 205–210 8337761 10.1016/0168-9525(93)90120-7

[bib55] Mabuchi K. Miya M. Azuma Y. Nishida M., 2007 Independent evolution of the specialized pharyngeal jaw apparatus in cichlid and labrid fishes. BMC Evol. Biol. 7: 10 17263894 10.1186/1471-2148-7-10 PMC1797158

[bib56] Manousaki T. Hull P. Kusche H. Machado-Schiaffino G. Franchini P., 2013 Parsing parallel evolution: ecological divergence and differential gene expression in thick-lipped Midas cichlid fishes of Nicaragua. Mol. Ecol. (in press) DOI: 10.1111/mec.12034 10.1111/mec.12034 23057963

[bib57] Matschiner M. Hanel R. Salzburger W., 2011 On the origin and trigger of the notothenioid adaptive radiation. PLoS ONE 6: e18911 21533117 10.1371/journal.pone.0018911 PMC3078932

[bib58] Meyer A., 1987 Phenotypic plasticity and heterochrony in *Cichlasoma managuense* (Pisces, Cichlidae) and their implications for speciation in cichlid fishes. Evolution 41: 1357–1369 10.1111/j.1558-5646.1987.tb02473.x 28563603

[bib59] Meyer A., 1990 a Ecological and evolutionary consequences of the trophic polymorphism in *Cichlasoma citrinellum* (Pisces: Cichlidae). Biol. J. Linn. Soc. Lond. 39: 279–299

[bib60] Meyer A., 1990 b Morphometrics and allometry in the trophically polymorphic cichlid fish, *Cichlasoma citrinellum*: alternative adaptations and ontogenetic changes in shape. J. Zool. (Lond.) 221: 237–260

[bib61] Meyer A., 1993 Phylogenetic relationships and evolutionary processes in East African cichlids. Trends Ecol. Evol. 8: 279–284 21236169 10.1016/0169-5347(93)90255-N

[bib62] Meyer A. Schartl M., 1999 Gene and genome duplications in vertebrates: the one-to-four (-to-eight in fish) rule and the evolution of novel gene functions. Curr. Opin. Cell Biol. 11: 699–704 10600714 10.1016/s0955-0674(99)00039-3

[bib63] Moyle L. C. Graham E. B., 2005 Genetics of hybrid incompatibility between *Lycopersicon esculentum* and *L. hirsutum*. Genetics 169: 355–373 15466436 10.1534/genetics.104.029546 PMC1448897

[bib64] Muller H., 1942 Isolating mechanisms, evolution and temperature. Biol. Symp. 6: 71–125

[bib66] Myburg A. A. Vogl C. Griffin A. R. Sederoff R. R. Whetten R. W., 2004 Genetics of postzygotic isolation in Eucalyptus: whole-genome analysis of barriers to introgression in a wide interspecific cross of *Eucalyptus grandis* and *E. globulus*. Genetics 166: 1405–1418 15082559 10.1534/genetics.166.3.1405 PMC1470765

[bib67] Nelson M. N. Moolhuijzen P. M. Boersma J. G. Chudy M. Lesniewska K., 2010 Aligning a new reference genetic map of *Lupinus angustifolius* with the genome sequence of the model legume, *Lotus japonicus*. DNA Res. 17: 73–83 20133394 10.1093/dnares/dsq001 PMC2853381

[bib68] Niehuis O. Judson A. K. Gadau J., 2008 Cytonuclear genic incompatibilities cause increased mortality in male F_2_ hybrids of *Nasonia giraulti* and *N. vitripennis*. Genetics 178: 413–426 18202384 10.1534/genetics.107.080523 PMC2206090

[bib69] Nosil P. Schluter D., 2011 The genes underlying the process of speciation. Trends Ecol. Evol. 26: 160–167 21310503 10.1016/j.tree.2011.01.001

[bib70] Parnell N. F. Hulsey C. D. Streelman J. T., 2012 The genetic basis of a complex functional system. Evolution 66: 3352–3366 23106702 10.1111/j.1558-5646.2012.01688.x PMC3490443

[bib71] Peterson B. K. Weber J. N. Kay E. H. Fisher H. S. Hoekstra H. E., 2012 Double digest RADseq: an inexpensive method for *de novo* SNP discovery and genotyping in model and non-model species. PLoS ONE 7: e37135 22675423 10.1371/journal.pone.0037135 PMC3365034

[bib72] Pfender W. Saha M. Johnson E. Slabaugh M., 2011 Mapping with RAD (restriction-site associated DNA) markers to rapidly identify QTL for stem rust resistance in *Lolium perenne*. Theor. Appl. Genet. 122: 1467–1480 21344184 10.1007/s00122-011-1546-3

[bib73] Phadnis N. Orr H. A., 2009 A single gene causes both male sterility and segregation distortion in *Drosophila* hybrids. Science 323: 376–379 19074311 10.1126/science.1163934 PMC2628965

[bib74] Ravi V. Venkatesh B., 2008 Rapidly evolving fish genomes and teleost diversity. Curr. Opin. Genet. Dev. 18: 544–550 19095434 10.1016/j.gde.2008.11.001

[bib75] Roberts R. B. Ser J. R. Kocher T. D., 2009 Sexual conflict resolved by invasion of a novel sex determiner in Lake Malawi cichlid fishes. Science 326: 998–1001 19797625 10.1126/science.1174705 PMC3174268

[bib96] Roberts R. B. Hu Y. Albertson R. C. Kocher T. D., 2011 Craniofacial divergence and ongoing adaptation via the hedgehog pathway. Proc. Natl. Acad. Sci. USA 108: 13194–13199 21788496 10.1073/pnas.1018456108 PMC3156204

[bib76] Rogers S. Bernatchez L., 2007 The genetic architecture of ecological speciation and the association with signatures of selection in natural lake whitefish (*Coregonus* sp. Salmonidae) species pairs. Mol. Biol. Evol. 24: 1423–1438 17404398 10.1093/molbev/msm066

[bib77] Rogers S. M. Isabel N. Bernatchez L., 2007 Linkage maps of the dwarf and normal lake whitefish (*Coregonus clupeaformis*) species complex and their hybrids reveal the genetic architecture of population divergence. Genetics 175: 375–398 17110497 10.1534/genetics.106.061457 PMC1774998

[bib78] Salzburger W., 2009 The interaction of sexually and naturally selected traits in the adaptive radiations of cichlid fishes. Mol. Ecol. 18: 169–185 18992003 10.1111/j.1365-294X.2008.03981.x

[bib79] Salzburger W. Meyer A., 2004 The species flocks of East African cichlid fishes: recent advances in molecular phylogenetics and population genetics. Naturwissenschaften 91: 277–290 15241604 10.1007/s00114-004-0528-6

[bib80] Salzburger W. Braasch I. Meyer A., 2007 Adaptive sequence evolution in a color gene involved in the formation of the characteristic egg-dummies of male haplochromine cichlid fishes. BMC Biol. 5: 51 18005399 10.1186/1741-7007-5-51 PMC2254590

[bib81] Sanetra M. Henning F. Fukamachi S. Meyer A., 2009 A microsatellite-based genetic linkage map of the cichlid fish, *Astatotilapia burtoni* (Teleostei): a comparison of genomic architectures among rapidly speciating cichlids. Genetics 182: 387–397 18757932 10.1534/genetics.108.089367 PMC2674835

[bib83] Stapley J. Reger J. Feulner P. G. D. Smadja C. Galindo J., 2010 Adaptation genomics: the next generation. Trends Ecol. Evol. 25: 705–712 20952088 10.1016/j.tree.2010.09.002

[bib84] Steinke D. Salzburger W. Meyer A., 2006 Novel relationships among ten fish model species revealed based on a phylogenomic analysis using ESTs. J. Mol. Evol. 62: 772–784 16752215 10.1007/s00239-005-0170-8

[bib86] Stiassny M. L. J. Meyer A., 1999 Cichlids of the Rift lakes. Sci. Am. 280: 64–69

[bib87] Streelman J. T. Albertson R. C., 2006 Evolution of novelty in the cichlid dentition. J. Exp. Zool. Part B 306: 216–226 10.1002/jez.b.21101 16555305

[bib88] Subramanian S. Denver D. R. Millar C. D. Heupink T. Aschrafi A., 2009 High mitogenomic evolutionary rates and time dependency. Trends Genet. 25: 482–486 19836098 10.1016/j.tig.2009.09.005

[bib89] Van Ooijen J., 2006 JoinMap 4. Software for the calculation of genetic linkage maps in experimental populations. Available at: http://www.kyazma.nl/index.php/mc.JoinMap. Accessed: November 28, 2012

[bib90] Vences M. Freyhof J. Sonnenberg R. Kosuch J. Veith M., 2001 Reconciling fossils and molecules: cenozoic divergence of cichlid fishes and the biogeography of Madagascar. J. Biogeogr. 28: 1091–1099

[bib91] Vivas R. McKaye K. R., 2001 Habitat selection, feeding ecology and fry survivorship in the *Amphilophus citrinellus* species complex in Lake Xiloá, Nicaragua. J. Aquacult. Aq. Sci. 9: 32–48

[bib92] Whitkus R., 1998 Genetics of adaptive radiation in Hawaiian and Cook Islands species of Tetramolopium (Asteraceae). II. Genetic linkage map and its implications for interspecific breeding barriers. Genetics 150: 1209–1216 9799272 10.1093/genetics/150.3.1209 PMC1460376

[bib93] Wilson A. B. Noack–Kunnmann K. Meyer A., 2000 Incipient speciation in sympatric Nicaraguan crater lake cichlid fishes: sexual selection *vs.* ecological diversification. Proc. Biol. Sci. 267: 2133–2141 11413624 10.1098/rspb.2000.1260 PMC1690797

[bib94] Wittbrodt J. Meyer A. Schartl M., 1998 More genes in fish? Bioessays 20: 511–512

