# Supplementary material for: A Hybrid Genetic Linkage Map of Two Ecologically and Morphologically Divergent Midas Cichlid Fishes (Amphilophus spp.) Obtained by Massively Parallel DNA Sequencing (ddRADSeq)
Source: G3 (Bethesda). 2013 Jan 1;3(1):65–74. doi: 10.1534/g3.112.003897 (PMC3538344; doi:10.1534/g3.112.003897)
Supplement: Supporting Information [file supp_3_1_65__index.html]

Supporting Information 

# A Hybrid Genetic Linkage Map of Two Ecologically and Morphologically Divergent Midas Cichlid Fishes (*Amphilophus* spp.) Obtained by Massively Parallel DNA Sequencing (ddRADSeq)

## Supporting Information for Recknagel *et al.*, 2013

**Files in this Data Supplement:**

- Supporting Information - Figure S1 and Tables S1-S5 (PDF, 208 KB)
- Figure S1 - Proportion of loci present in number (N) of individuals (PDF, 105 KB)
- Table S1 - P1 and P2 adapter sequences (PDF, 58 KB)
- Table S2 - Individual's sequenced genomic output (PDF, 54 KB)
- Table S4 - Length and number of markers per linkage group and average spacing distance between markers (PDF, 48 KB)
- Table S5 - RAD markers used in the comparative analyses and mapped to tilapia, stickleback and medaka (PDF, 114 KB)
- Table S3 - RAD marker order, position on linkage group, sequences and genotypes (.xlsx, 1.1 MB)
