# Supplementary material for: A Hybrid Genetic Linkage Map of Two Ecologically and Morphologically Divergent Midas Cichlid Fishes (Amphilophus spp.) Obtained by Massively Parallel DNA Sequencing (ddRADSeq)
Source: G3 (Bethesda). 2013 Jan 1;3(1):65–74. doi: 10.1534/g3.112.003897 (PMC3538344; doi:10.1534/g3.112.003897)
Supplement: Supporting Information [file supp_3.1.65_003897SI.pdf]

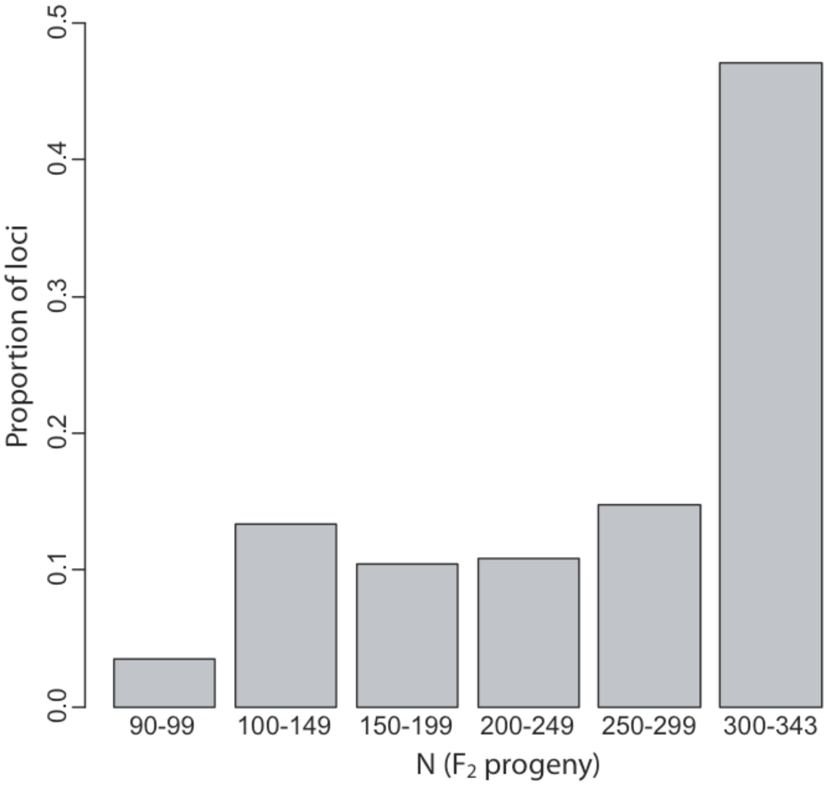

Figure S1 Proportion of loci present in number (N) of individuals

Table S1 P1 and P2 adapter sequences

ID	Sequence
KE_P1thioFwAACCC	AATGATACGGCGACCACCGAGATCTACACTCTTTCCCTACACGACGCTCTTCCGATCTAACCCTGCA
KE_P1thioFwAAGGG	AATGATACGGCGACCACCGAGATCTACACTCTTTCCCTACACGACGCTCTTCCGATCTAAGGGTGCA
KE_P1thioFwACTGC	AATGATACGGCGACCACCGAGATCTACACTCTTTCCCTACACGACGCTCTTCCGATCTACTGCTGCA
KE_P1thioFwAGAGT	AATGATACGGCGACCACCGAGATCTACACTCTTTCCCTACACGACGCTCTTCCGATCTAGAGTTGCA
KE_P1thioFwAGCTG	AATGATACGGCGACCACCGAGATCTACACTCTTTCCCTACACGACGCTCTTCCGATCTAGCTGTGCA
KE_P1thioFwAGGAC	AATGATACGGCGACCACCGAGATCTACACTCTTTCCCTACACGACGCTCTTCCGATCTAGGACTGCA
KE_P1thioFwAGTCA	AATGATACGGCGACCACCGAGATCTACACTCTTTCCCTACACGACGCTCTTCCGATCTAGTCATGCA
KE_P1thioFwATATC	AATGATACGGCGACCACCGAGATCTACACTCTTTCCCTACACGACGCTCTTCCGATCTATATCTGCA
KE_P1thioFwATCGA	AATGATACGGCGACCACCGAGATCTACACTCTTTCCCTACACGACGCTCTTCCGATCTATCGATGCA
KE_P1thioFwATGCT	AATGATACGGCGACCACCGAGATCTACACTCTTTCCCTACACGACGCTCTTCCGATCTATGCTTGCA
KE_P1thioFwATTAG	AATGATACGGCGACCACCGAGATCTACACTCTTTCCCTACACGACGCTCTTCCGATCTATTAGTGCA
KE_P1thioFwCAACT	AATGATACGGCGACCACCGAGATCTACACTCTTTCCCTACACGACGCTCTTCCGATCTCAACTTGCA
KE_P1thioFwCACAG	AATGATACGGCGACCACCGAGATCTACACTCTTTCCCTACACGACGCTCTTCCGATCTCACAGTGCA
KE_P1thioFwCAGTC	AATGATACGGCGACCACCGAGATCTACACTCTTTCCCTACACGACGCTCTTCCGATCTCAGTCTGCA
KE_P1thioFwCATGA	AATGATACGGCGACCACCGAGATCTACACTCTTTCCCTACACGACGCTCTTCCGATCTCATGATGCA
KE_P1thioFwCCAAC	AATGATACGGCGACCACCGAGATCTACACTCTTTCCCTACACGACGCTCTTCCGATCTCCAACTGCA
KE_P1thioFwCCCCA	AATGATACGGCGACCACCGAGATCTACACTCTTTCCCTACACGACGCTCTTCCGATCTCCCCATGCA
KE_P1thioFwCCGGT	AATGATACGGCGACCACCGAGATCTACACTCTTTCCCTACACGACGCTCTTCCGATCTCCGGTTGCA
KE_P1thioFwCCTTG	AATGATACGGCGACCACCGAGATCTACACTCTTTCCCTACACGACGCTCTTCCGATCTCCTTGTGCA
KE_P1thioFwCGATA	AATGATACGGCGACCACCGAGATCTACACTCTTTCCCTACACGACGCTCTTCCGATCTCGATATGCA
KE_P1thioFwCGCGC	AATGATACGGCGACCACCGAGATCTACACTCTTTCCCTACACGACGCTCTTCCGATCTCGCGCTGCA
KE_P1thioFwCGGCG	AATGATACGGCGACCACCGAGATCTACACTCTTTCCCTACACGACGCTCTTCCGATCTCGGCGTGCA
KE_P1thioFwCGTAT	AATGATACGGCGACCACCGAGATCTACACTCTTTCCCTACACGACGCTCTTCCGATCTCGTATTGCA
KE_P1thioFwCTAGG	AATGATACGGCGACCACCGAGATCTACACTCTTTCCCTACACGACGCTCTTCCGATCTCTAGGTGCA
KE_P1thioFwCTCTT	AATGATACGGCGACCACCGAGATCTACACTCTTTCCCTACACGACGCTCTTCCGATCTCTCTTTGCA
KE_P1thioFwCTTCC	AATGATACGGCGACCACCGAGATCTACACTCTTTCCCTACACGACGCTCTTCCGATCTCTTCCTGCA
KE_P1thioFwGAAGC	AATGATACGGCGACCACCGAGATCTACACTCTTTCCCTACACGACGCTCTTCCGATCTGAAGCTGCA
KE_P1thioFwGACTA	AATGATACGGCGACCACCGAGATCTACACTCTTTCCCTACACGACGCTCTTCCGATCTGACTATGCA
KE_P1thioFwGAGAT	AATGATACGGCGACCACCGAGATCTACACTCTTTCCCTACACGACGCTCTTCCGATCTGAGATTGCA
KE_P1thioFwGATCG	AATGATACGGCGACCACCGAGATCTACACTCTTTCCCTACACGACGCTCTTCCGATCTGATCGTGCA
KE_P1thioFwGCATT	AATGATACGGCGACCACCGAGATCTACACTCTTTCCCTACACGACGCTCTTCCGATCTGCATTTGCA
KE_P1thioFwGCCGG	AATGATACGGCGACCACCGAGATCTACACTCTTTCCCTACACGACGCTCTTCCGATCTGCCGGTGCA
KE_P1thioFwGCGCC	AATGATACGGCGACCACCGAGATCTACACTCTTTCCCTACACGACGCTCTTCCGATCTGCGCCTGCA
KE_P1thioFwGCTAA	AATGATACGGCGACCACCGAGATCTACACTCTTTCCCTACACGACGCTCTTCCGATCTGCTAATGCA
KE_P1thioFwGGAAG	AATGATACGGCGACCACCGAGATCTACACTCTTTCCCTACACGACGCTCTTCCGATCTGGAAGTGCA
KE_P1thioFwGGCCT	AATGATACGGCGACCACCGAGATCTACACTCTTTCCCTACACGACGCTCTTCCGATCTGGCCTTGCA
KE_P1thioFwGGGGA	AATGATACGGCGACCACCGAGATCTACACTCTTTCCCTACACGACGCTCTTCCGATCTGGGGATGCA
KE_P1thioFwGGTTC	AATGATACGGCGACCACCGAGATCTACACTCTTTCCCTACACGACGCTCTTCCGATCTGGTTCTGCA

KE_P1thioFwGTACA	AATGATACGGCGACCACCGAGATCTACACTCTTTCCCTACACGACGCTCTTCCGATCTGTACATGCA
KE_P1thioFwGTCAC	AATGATACGGCGACCACCGAGATCTACACTCTTTCCCTACACGACGCTCTTCCGATCTGTCACTGCA
KE_P1thioFwGTGTG	AATGATACGGCGACCACCGAGATCTACACTCTTTCCCTACACGACGCTCTTCCGATCTGTGTGTGCA
KE_P1thioFwGTTGT	AATGATACGGCGACCACCGAGATCTACACTCTTTCCCTACACGACGCTCTTCCGATCTGTTGTTGCA
KE_P1thioFwTTAAT	AATGATACGGCGACCACCGAGATCTACACTCTTTCCCTACACGACGCTCTTCCGATCTTTAATTGCA
KE_P1thioFwTTCCG	AATGATACGGCGACCACCGAGATCTACACTCTTTCCCTACACGACGCTCTTCCGATCTTTCCGTGCA
KE_P1thioFwAAAAA	AATGATACGGCGACCACCGAGATCTACACTCTTTCCCTACACGACGCTCTTCCGATCTAAAAATGCA
KE_P1thioFwAATTT	AATGATACGGCGACCACCGAGATCTACACTCTTTCCCTACACGACGCTCTTCCGATCTAATTTTGCA
KE_P1thioFwACACG	AATGATACGGCGACCACCGAGATCTACACTCTTTCCCTACACGACGCTCTTCCGATCTACACGTGCA
KE_P1thioFwACCAT	AATGATACGGCGACCACCGAGATCTACACTCTTTCCCTACACGACGCTCTTCCGATCTACCATTGCA
KE_P1thioFwACGTA	AATGATACGGCGACCACCGAGATCTACACTCTTTCCCTACACGACGCTCTTCCGATCTACGTATGCA
KE_P1thioFwTTTTT	AATGATACGGCGACCACCGAGATCTACACTCTTTCCCTACACGACGCTCTTCCGATCTTTTTTTGCA
KE_P1PthioRvAACCC	GGGTTAGATCGGAAGAGCGTCGTGTAGGGAAAGAGTGTAGATCTCGGTGGTCGCCGTATCATT
KE_P1PthioRvAAGGG	CCCTTAGATCGGAAGAGCGTCGTGTAGGGAAAGAGTGTAGATCTCGGTGGTCGCCGTATCATT
KE_P1PthioRvACTGC	GCAGTAGATCGGAAGAGCGTCGTGTAGGGAAAGAGTGTAGATCTCGGTGGTCGCCGTATCATT
KE_P1PthioRvAGAGT	ACTCTAGATCGGAAGAGCGTCGTGTAGGGAAAGAGTGTAGATCTCGGTGGTCGCCGTATCATT
KE_P1PthioRvAGCTG	CAGCTAGATCGGAAGAGCGTCGTGTAGGGAAAGAGTGTAGATCTCGGTGGTCGCCGTATCATT
KE_P1PthioRvAGGAC	GTCCTAGATCGGAAGAGCGTCGTGTAGGGAAAGAGTGTAGATCTCGGTGGTCGCCGTATCATT
KE_P1PthioRvAGTCA	TGACTAGATCGGAAGAGCGTCGTGTAGGGAAAGAGTGTAGATCTCGGTGGTCGCCGTATCATT
KE_P1PthioRvATATC	GATATAGATCGGAAGAGCGTCGTGTAGGGAAAGAGTGTAGATCTCGGTGGTCGCCGTATCATT
KE_P1PthioRvATCGA	TCGATAGATCGGAAGAGCGTCGTGTAGGGAAAGAGTGTAGATCTCGGTGGTCGCCGTATCATT
KE_P1PthioRvATGCT	AGCATAGATCGGAAGAGCGTCGTGTAGGGAAAGAGTGTAGATCTCGGTGGTCGCCGTATCATT
KE_P1PthioRvATTAG	CTAATAGATCGGAAGAGCGTCGTGTAGGGAAAGAGTGTAGATCTCGGTGGTCGCCGTATCATT
KE_P1PthioRvCAACT	AGTTGAGATCGGAAGAGCGTCGTGTAGGGAAAGAGTGTAGATCTCGGTGGTCGCCGTATCATT
KE_P1PthioRvCACAG	CTGTGAGATCGGAAGAGCGTCGTGTAGGGAAAGAGTGTAGATCTCGGTGGTCGCCGTATCATT
KE_P1PthioRvCAGTC	GACTGAGATCGGAAGAGCGTCGTGTAGGGAAAGAGTGTAGATCTCGGTGGTCGCCGTATCATT
KE_P1PthioRvCATGA	TCATGAGATCGGAAGAGCGTCGTGTAGGGAAAGAGTGTAGATCTCGGTGGTCGCCGTATCATT
KE_P1PthioRvCCAAC	GTTGGAGATCGGAAGAGCGTCGTGTAGGGAAAGAGTGTAGATCTCGGTGGTCGCCGTATCATT
KE_P1PthioRvCCCCA	TGGGGAGATCGGAAGAGCGTCGTGTAGGGAAAGAGTGTAGATCTCGGTGGTCGCCGTATCATT
KE_P1PthioRvCCGGT	ACCGGAGATCGGAAGAGCGTCGTGTAGGGAAAGAGTGTAGATCTCGGTGGTCGCCGTATCATT
KE_P1PthioRvCCTTG	CAAGGAGATCGGAAGAGCGTCGTGTAGGGAAAGAGTGTAGATCTCGGTGGTCGCCGTATCATT
KE_P1PthioRvCGATA	TATCGAGATCGGAAGAGCGTCGTGTAGGGAAAGAGTGTAGATCTCGGTGGTCGCCGTATCATT
KE_P1PthioRvCGCGC	GCGCGAGATCGGAAGAGCGTCGTGTAGGGAAAGAGTGTAGATCTCGGTGGTCGCCGTATCATT
KE_P1PthioRvCGGCG	CGCCGAGATCGGAAGAGCGTCGTGTAGGGAAAGAGTGTAGATCTCGGTGGTCGCCGTATCATT
KE_P1PthioRvCGTAT	ATACGAGATCGGAAGAGCGTCGTGTAGGGAAAGAGTGTAGATCTCGGTGGTCGCCGTATCATT
KE_P1PthioRvCTAGG	CCTAGAGATCGGAAGAGCGTCGTGTAGGGAAAGAGTGTAGATCTCGGTGGTCGCCGTATCATT
KE_P1PthioRvCTCTT	AAGAGAGATCGGAAGAGCGTCGTGTAGGGAAAGAGTGTAGATCTCGGTGGTCGCCGTATCATT
KE_P1PthioRvCTTCC	GGAAGAGATCGGAAGAGCGTCGTGTAGGGAAAGAGTGTAGATCTCGGTGGTCGCCGTATCATT
KE_P1PthioRvGAAGC	GCTTCAGATCGGAAGAGCGTCGTGTAGGGAAAGAGTGTAGATCTCGGTGGTCGCCGTATCATT
KE_P1PthioRvGACTA	TAGTCAGATCGGAAGAGCGTCGTGTAGGGAAAGAGTGTAGATCTCGGTGGTCGCCGTATCATT

KE_P1PthioRvGAGAT	ATCTCAGATCGGAAGAGCGTCGTGTAGGGAAAGAGTGTAGATCTCGGTGGTCGCCGTATCATT
KE_P1PthioRvGATCG	CGATCAGATCGGAAGAGCGTCGTGTAGGGAAAGAGTGTAGATCTCGGTGGTCGCCGTATCATT
KE_P1PthioRvGCATT	AATGCAGATCGGAAGAGCGTCGTGTAGGGAAAGAGTGTAGATCTCGGTGGTCGCCGTATCATT
KE_P1PthioRvGCCGG	CCGGCAGATCGGAAGAGCGTCGTGTAGGGAAAGAGTGTAGATCTCGGTGGTCGCCGTATCATT
KE_P1PthioRvGCGCC	GGCGCAGATCGGAAGAGCGTCGTGTAGGGAAAGAGTGTAGATCTCGGTGGTCGCCGTATCATT
KE_P1PthioRvGCTAA	TTAGCAGATCGGAAGAGCGTCGTGTAGGGAAAGAGTGTAGATCTCGGTGGTCGCCGTATCATT
KE_P1PthioRvGGAAG	CTTCCAGATCGGAAGAGCGTCGTGTAGGGAAAGAGTGTAGATCTCGGTGGTCGCCGTATCATT
KE_P1PthioRvGGCCT	AGGCCAGATCGGAAGAGCGTCGTGTAGGGAAAGAGTGTAGATCTCGGTGGTCGCCGTATCATT
KE_P1PthioRvGGGGA	TCCCCAGATCGGAAGAGCGTCGTGTAGGGAAAGAGTGTAGATCTCGGTGGTCGCCGTATCATT
KE_P1PthioRvGGTTC	GAACCAGATCGGAAGAGCGTCGTGTAGGGAAAGAGTGTAGATCTCGGTGGTCGCCGTATCATT
KE_P1PthioRvGTACA	TGTACAGATCGGAAGAGCGTCGTGTAGGGAAAGAGTGTAGATCTCGGTGGTCGCCGTATCATT
KE_P1PthioRvGTCAC	GTGACAGATCGGAAGAGCGTCGTGTAGGGAAAGAGTGTAGATCTCGGTGGTCGCCGTATCATT
KE_P1PthioRvGTGTG	CACACAGATCGGAAGAGCGTCGTGTAGGGAAAGAGTGTAGATCTCGGTGGTCGCCGTATCATT
KE_P1PthioRvGTTGT	ACAACAGATCGGAAGAGCGTCGTGTAGGGAAAGAGTGTAGATCTCGGTGGTCGCCGTATCATT
KE_P1PthioRvTTAAT	ATTAAAGATCGGAAGAGCGTCGTGTAGGGAAAGAGTGTAGATCTCGGTGGTCGCCGTATCATT
KE_P1PthioRvTTCCG	CGGAAAGATCGGAAGAGCGTCGTGTAGGGAAAGAGTGTAGATCTCGGTGGTCGCCGTATCATT
KE_P1PthioRvAAAAA	TTTTTAGATCGGAAGAGCGTCGTGTAGGGAAAGAGTGTAGATCTCGGTGGTCGCCGTATCATT
KE_P1PthioRvAATTT	AAATTAGATCGGAAGAGCGTCGTGTAGGGAAAGAGTGTAGATCTCGGTGGTCGCCGTATCATT
KE_P1PthioRvACACG	CGTGTAGATCGGAAGAGCGTCGTGTAGGGAAAGAGTGTAGATCTCGGTGGTCGCCGTATCATT
KE_P1PthioRvACCAT	ATGGTAGATCGGAAGAGCGTCGTGTAGGGAAAGAGTGTAGATCTCGGTGGTCGCCGTATCATT
KE_P1PthioRvACGTA	TACGTAGATCGGAAGAGCGTCGTGTAGGGAAAGAGTGTAGATCTCGGTGGTCGCCGTATCATT
KE_P1PthioRvTTTTT	AAAAAAGATCGGAAGAGCGTCGTGTAGGGAAAGAGTGTAGATCTCGGTGGTCGCCGTATCATT
KE_P2PhoFwCGpair	CGAGATCGGAAGAGCGGTTCAGCAGGAATGCCGAGACCGATCAGAACAA
KE_P2thioRvpair	CAAGCAGAAGACGGCATACGAGATCGGTCTCGGCATTCCTGCTGAACCGCTCTTCCGATCT

Identification (=ID) defines adapter type (P1 or P2), modification (phosphorothioate bond = thio, 5’ Phosphorylation = P), direction (Fw = forward, RV = reverse), and 5 base barcode.

Table S2 Individual's sequenced genomic output

	N	N loci	N loci polymorphic	N SNPs	SNP frequency	coverage
A. zaliosus, male	1	142740	9531	12764	0.089	15.6x
A. astorquii, female	1	139109	8150	11172	0.080	15.0x
F1 hybrids	2	140925	9336	11968	0.090	14.0x
F2 hybrids	343	36851	1633	2257	0.061	15.2x

Note that in the parents and F_1 hybrids a higher gel size range was selected than for F_2 individuals and therefore they exhibit a higher number of loci.

Table S3 RAD marker order, position on linkage group, sequences and genotypes

Available for download at http://www.g3journal.org/lookup/suppl/doi:10.1534/g3.112.003897/-/DC1. The number in 'SNPs' refers to the position of the SNP in the RAD marker sequence and is followed by the nucleotide which is substituted. F_2 offspring genotypes are coded as a=homozygous for *A. astorquii* alleles, h=heterozygous, b=homozygous for *A. zaliosus*. SD = segregation distortion; * P<0.05, * P<0.01, *** P<0.001; Synteny = number of synteny analyses in which a marker was deployed.

Table S4   Length and number of markers per linkage group and average spacing distance between markers

LG	Spacing [cM]	Markers [N]	Length [cM]
1	1.61	49	77.1
2	2.56	28	69.2
3	2.16	33	69.1
4	1.44	49	69.0
5	1.25	54	66.1
6	2.18	31	65.4
7	3.27	21	65.4
8	2.84	24	65.3
9	3.32	20	63.0
10	1.53	42	62.9
11	1.67	38	61.9
12	2.18	29	61.1
13	1.89	33	60.4
14	2.99	21	59.8
15	1.42	43	59.7
16	4.57	14	59.4
17	1.36	44	58.4
18	1.87	32	58.0
19	2.04	29	57.1
20	6.07	10	54.6
21	1.65	34	54.4
22	2.69	21	53.7
23	1.03	51	51.5
24	1.27	3	2.5
25	1.99	2	2.0
**Average**	1.95	30.2	59.4
**Total**	-	755	1426.9

Table S5 RAD markers used in the comparative analyses and mapped to tilapia, stickleback and medaka

RAD-ID	Midas LG	Mapped to:	LG	LG size	E-value	Start [bp]	Length [bp]
36474	1	tilapia	LG16-21	34890008	1.00E-24	27352408	106
43465	1	tilapia	LG3	19325363	2.00E-26	18187493	109
100846	1	tilapia	LG3	19325363	2.00E-26	12853769	109
16458	1	tilapia	LG3	19325363	3.00E-41	11674415	110
92986	1	tilapia	LG3	19325363	2.00E-42	10094525	96
94064	1	tilapia	LG3	19325363	3.00E-16	10064159	80
45072	1	tilapia	LG3	19325363	7.00E-36	7854642	93
31789	1	tilapia	LG3	19325363	1.00E-15	5068289	96
66818	2	tilapia	LG6	36725243	3.00E-41	33567821	110
17185	2	tilapia	LG6	36725243	4.00E-25	31586102	95
24490	2	tilapia	LG6	36725243	3.00E-35	25607155	108
16242	2	tilapia	LG6	36725243	3.00E-41	28181098	110
109951	2	tilapia	LG6	36725243	1.00E-24	35791579	62
76409	2	tilapia	LG6	36725243	2.00E-48	7839121	110
55453	2	tilapia	LG6	36725243	2.00E-39	7709326	99
98129	2	tilapia	LG6	36725243	3.00E-16	11650745	72
49108	2	tilapia	LG6	36725243	2.00E-48	11279958	110
41452	3	tilapia	LG14	34191023	2.00E-26	32476326	93
40968	3	tilapia	LG14	34191023	2.00E-27	33214185	83
7637	3	tilapia	LG14	34191023	3.00E-32	31227075	83
63920	3	tilapia	LG14	34191023	1.00E-31	3626355	110
99911	3	tilapia	LG14	34191023	2.00E-33	10534804	97
83872	3	tilapia	LG14	34191023	1.00E-28	6925433	89
6497	3	tilapia	LG14	34191023	4.00E-31	27684349	93
5041	3	tilapia	LG3	19325363	1.00E-15	12923096	71
20407	4	tilapia	LG5	37389089	4.00E-34	31342438	110
5853	4	tilapia	LG5	37389089	6.00E-27	13957074	70
84930	4	tilapia	LG5	37389089	1.00E-34	13964233	95
17788	4	tilapia	LG5	37389089	4.00E-31	7297095	97
70499	4	tilapia	LG6	36725243	1.00E-15	31184138	67
100566	5	tilapia	LG23	20779993	1.00E-55	7153106	110
101011	5	tilapia	scaffold3	NA	9.00E-20	3004164	98
124145	5	tilapia	LG4	28679955	7.00E-33	2864401	104
107800	5	tilapia	LG23	20779993	1.00E-31	13864662	110
70700	5	tilapia	LG23	20779993	3.00E-19	15261246	57
105100	5	tilapia	LG23	20779993	5.00E-18	18390222	75

25979	5	tilapia	LG23	20779993	1.00E-18	20545645	84
18719	5	tilapia	scaffold4	NA	5.00E-18	217438	71
26115	5	tilapia	scaffold4	NA	6.00E-27	1258943	74
46405	5	tilapia	LG2	25048291	9.00E-20	11839398	82
20172	5	tilapia	LG17	31749960	2.00E-36	2679164	110
14319	5	tilapia	scaffold131	NA	1.00E-24	351651	110
15733	5	tilapia	scaffold131	NA	1.00E-25	482104	84
33213	5	tilapia	LG6	36725243	1.00E-18	25758117	76
134694	5	tilapia	scaffold67	NA	1.00E-25	409235	104
116962	6	tilapia	LG13	32787261	1.00E-18	13164943	68
22729	6	tilapia	LG11	33447472	1.00E-18	16371622	60
11412	6	tilapia	LG11	33447472	1.00E-25	17320254	95
75436	6	tilapia	LG11	33447472	1.00E-25	11552185	92
87932	6	tilapia	LG11	33447472	1.00E-43	12025784	110
44241	6	tilapia	LG11	33447472	4.00E-31	12586794	108
34216	6	tilapia	LG11	33447472	1.00E-40	26837191	109
28307	7	tilapia	scaffold66	NA	6.00E-30	773981	107
79933	7	tilapia	LG20	31470686	3.00E-16	9262740	52
66611	7	tilapia	LG20	31470686	1.00E-18	12387108	96
38956	7	tilapia	LG20	31470686	8.00E-48	26889516	109
27002	7	tilapia	LG20	31470686	1.00E-24	30315062	82
50804	8	tilapia	LG1	31194787	2.00E-36	9414368	110
2276	9	tilapia	LG7	51042256	2.00E-29	33398893	110
62272	9	tilapia	LG7	51042256	2.00E-33	33163211	89
102680	9	tilapia	LG7	51042256	1.00E-15	25377123	83
67458	9	tilapia	LG7	51042256	3.00E-19	14336881	85
35953	10	tilapia	LG15	26684556	2.00E-26	9399831	69
61040	10	tilapia	LG6	36725243	1.00E-15	9454319	51
18661	10	tilapia	LG15	26684556	2.00E-17	25997391	102
37971	10	tilapia	LG15	26684556	2.00E-48	24044802	106
130058	10	tilapia	LG15	26684556	1.00E-55	22676238	110
3820	10	tilapia	LG15	26684556	6.00E-30	19826960	103
57690	10	tilapia	LG15	26684556	2.00E-42	14597823	108
19144	10	tilapia	LG15	26684556	5.00E-46	15270678	110
40973	10	tilapia	LG15	26684556	4.00E-22	14895757	110
31355	10	tilapia	LG15	26684556	4.00E-22	14330383	110
135135	11	tilapia	LG8-24	29447820	9.00E-20	16653790	78
88334	11	tilapia	LG2	25048291	6.00E-30	23221520	111
18172	11	tilapia	LG2	25048291	1.00E-46	16511820	107
2566	11	tilapia	LG2	25048291	7.00E-39	15288422	102

47254	11	tilapia	LG2	25048291	1.00E-18	10444085	84
7828	11	tilapia	LG2	25048291	3.00E-35	10374142	100
82738	12	tilapia	LG10	17092887	3.00E-16	6778356	84
20338	12	tilapia	scaffold8	NA	2.00E-33	650097	108
50896	12	tilapia	scaffold10	NA	4.00E-37	1680258	103
72849	13	tilapia	LG19	27159252	2.00E-39	5471797	107
13074	13	Tilapia	LG19	27159252	6.00E-27	3912948	82
43690	13	tilapia	LG19	27159252	1.00E-25	3114780	96
138263	13	tilapia	LG19	27159252	9.00E-20	2186973	98
68249	13	tilapia	LG19	27159252	6.00E-27	1322595	82
35488	13	tilapia	LG19	27159252	1.00E-18	19391465	88
25842	13	tilapia	LG19	27159252	7.00E-33	25935291	104
57774	13	tilapia	LG19	27159252	2.00E-17	24976001	70
120000	13	tilapia	scaffold236	NA	1.00E-43	261485	106
67135	14	tilapia	LG9	20956653	1.00E-24	8263710	98
124232	14	tilapia	LG9	20956653	2.00E-45	4905076	109
86011	14	tilapia	LG9	20956653	9.00E-20	1994927	86
102811	15	tilapia	LG12	34679706	2.00E-17	34340099	54
60944	15	tilapia	LG12	34679706	5.00E-18	29643656	91
27544	15	tilapia	LG12	34679706	2.00E-17	33221583	54
103035	15	tilapia	LG12	34679706	4.00E-31	4463867	69
89074	15	tilapia	LG12	34679706	2.00E-20	7390764	107
42654	15	tilapia	LG12	34679706	5.00E-46	12475370	110
49461	15	tilapia	LG12	34679706	2.00E-30	12999594	91
52449	15	tilapia	LG12	34679706	4.00E-34	21395792	110
110339	15	tilapia	LG12	34679706	8.00E-17	22548755	69
49654	15	tilapia	LG12	34679706	5.00E-18	24279788	91
92921	16	tilapia	scaffold30	NA	2.00E-33	983110	105
117088	17	tilapia	LG16-21	34890008	2.00E-39	24506599	107
35124	17	tilapia	LG16-21	34890008	6.00E-30	20371064	111
107705	17	tilapia	LG16-21	34890008	2.00E-20	10242661	107
95987	18	tilapia	LG22	26410405	2.00E-17	17476441	87
68597	18	tilapia	LG1	31194787	2.00E-17	1956352	97
60870	18	tilapia	LG22	26410405	1.00E-34	7337873	95
29586	18	tilapia	LG22	26410405	9.00E-20	6392193	58
78539	18	tilapia	LG22	26410405	7.00E-39	1725117	98
107830	19	tilapia	LG18	26198306	1.00E-15	1648104	99
63950	19	tilapia	LG18	26198306	6.00E-30	4430031	103
95865	19	tilapia	LG18	26198306	6.00E-27	7113536	90
25517	19	tilapia	LG18	26198306	4.00E-22	6917050	90

67084	19	tilapia	LG18	26198306	3.00E-50	8010479	109
26271	19	tilapia	LG18	26198306	2.00E-30	10395195	112
3078	19	tilapia	LG18	26198306	5.00E-18	11747611	51
34529	19	tilapia	LG18	26198306	1.00E-25	14013552	104
122299	19	tilapia	LG18	26198306	8.00E-51	22828155	110
43716	20	tilapia	LG13	32787261	3.00E-16	16538607	100
4469	20	tilapia	LG13	32787261	2.00E-30	11764859	108
132664	20	tilapia	LG13	32787261	6.00E-27	10199359	106
26739	20	tilapia	LG13	32787261	6.00E-27	10189582	78
21036	21	tilapia	LG17	31749960	3.00E-19	15765488	77
4370	21	tilapia	LG17	31749960	2.00E-36	16676140	110
14574	21	tilapia	LG17	31749960	3.00E-32	18448345	111
48454	21	tilapia	LG17	31749960	9.00E-20	18490652	78
69372	21	tilapia	LG17	31749960	1.00E-24	23733486	78
134952	21	tilapia	LG17	31749960	1.00E-18	8486526	60
61799	21	tilapia	LG17	31749960	2.00E-33	6985120	85
24976	21	tilapia	LG17	31749960	6.00E-21	6772819	68
20103	21	tilapia	LG20	31470686	2.00E-29	17556931	110
24087	22	tilapia	LG8-24	29447820	3.00E-32	7561884	110
37195	22	tilapia	LG9	20956653	1.00E-15	16088012	112
72695	22	tilapia	LG8-24	29447820	5.00E-40	23238281	104
83435	23	tilapia	scaffold2	NA	2.00E-23	2314070	108
50756	23	tilapia	LG8-24	29447820	2.00E-17	16653790	78
72264	23	tilapia	scaffold2	NA	1.00E-37	3309019	108
99870	23	tilapia	LG18	26198306	3.00E-16	23778455	56
102568	23	tilapia	scaffold2	NA	9.00E-20	2781012	54
14690	23	tilapia	scaffold2	NA	3.00E-32	1982515	116
28081	23	tilapia	scaffold2	NA	2.00E-23	40130	116
134769	23	tilapia	scaffold13	NA	1.00E-37	1610515	96
45048	23	tilapia	scaffold13	NA	2.00E-17	911984	102
50742	23	tilapia	scaffold2	NA	4.00E-22	3725596	62
44613	23	tilapia	scaffold37	NA	5.00E-18	186882	91
138376	23	tilapia	scaffold37	NA	1.00E-24	750203	110
67271	23	tilapia	scaffold35	NA	2.00E-27	489624	107
122919	23	tilapia	scaffold1	NA	4.00E-31	7541858	101
26368	23	tilapia	LG15	26684556	2.00E-20	1613651	87
111928	23	tilapia	scaffold1	NA	2.00E-20	5751988	55
88889	23	tilapia	scaffold1	NA	6.00E-21	4930700	100
92986	1	stickleback	LG7	27937443	1.00E-29	5268693	82
24490	2	stickleback	LG9	20249479	7.00E-19	11066750	84

16242	2	stickleback	LG9	20249479	8.00E-31	14844617	104
41452	3	stickleback	LG1	28185914	3.00E-21	15989330	104
63761	3	stickleback	LG11	16706052	7.00E-16	2155417	59
20407	4	stickleback	LG17	14603141	1.00E-11	3882191	44
137502	4	stickleback	scaffold161	158182	2.00E-16	5752	52
100566	5	stickleback	LG13	19368704	2.00E-46	2981141	110
124145	5	stickleback	LG13	19368704	5.00E-23	4597623	99
70700	5	stickleback	LG13	19368704	2.00E-13	17013080	51
15733	5	stickleback	LG13	19368704	4.00E-17	6347398	69
11412	6	stickleback	LG20	19732071	4.00E-14	10783875	52
75436	6	stickleback	LG20	19732071	4.00E-11	6554303	87
38956	7	stickleback	LG7	18401067	1.00E-17	10051916	78
27002	7	stickleback	LG7	18401067	2.00E-28	7500971	88
1438	8	stickleback	LG1	28185914	7.00E-19	7151749	84
50804	8	stickleback	LG2	23295652	2.00E-16	9015372	52
3820	10	stickleback	LG18	16282716	4.00E-20	15514611	98
57690	10	stickleback	LG18	16282716	4.00E-20	8245539	78
88334	11	stickleback	LG4	32632948	3.00E-24	14020516	85
18172	11	stickleback	LG4	32632948	8.00E-28	9477753	107
72849	13	stickleback	LG15	16198764	5.00E-23	8839077	99
13074	13	stickleback	LG15	16198764	2.00E-22	7264942	62
75998	13	stickleback	LG18	16282716	1.00E-17	189633	90
120000	13	stickleback	scaffold84	563433	2.00E-34	56700	110
79799	14	stickleback	LG2	23295652	1.00E-11	6873776	44
86011	14	stickleback	scaffold37	2648413	4.00E-11	2017509	43
27654	15	stickleback	LG13	20083130	6.00E-13	19696724	110
92921	16	stickleback	LG19	20240660	1.00E-14	8048874	81
18097	17	stickleback	LG14	15246461	4.00E-17	7950866	49
35783	18	stickleback	LG19	20240660	1.00E-17	1908224	66
29586	18	stickleback	LG10	15657440	3.00E-18	1653488	59
67084	19	stickleback	LG3	16798506	2.00E-13	7194870	51
3078	19	stickleback	LG3	16798506	6.00E-13	1040262	50
4370	21	stickleback	LG4	32632948	2.00E-13	32139127	95
134952	21	stickleback	LG4	32632948	1.00E-20	24701738	59
24976	21	stickleback	LG4	32632948	1.00E-14	24625077	65
72264	23	stickleback	scaffold74	682422	3.00E-15	404572	110
102568	23	stickleback	scaffold74	682422	6.00E-13	497533	54
14690	23	stickleback	scaffold868	8898	2.00E-16	1513	64
67271	23	stickleback	LG1	28185914	7.00E-22	22922476	109
122919	23	stickleback	LG1	28185914	2.00E-16	23030100	104

88889	23	stickleback	LG1	28185914	4.00E-11	24446156	63
120613	23	stickleback	scaffold37	2648413	4.00E-17	1572885	101
21428	2	medaka	LG14	33607196	3.00E-19	22450795	57
41452	3	medaka	LG13	33409148	7.00E-11	3373606	83
63761	3	medaka	LG2	31118443	2.00E-26	18951700	105
26388	3	medaka	LG2	31118443	3.00E-22	14124612	102
137502	4	medaka	LG10	27595823	1.00E-15	8274156	83
13782	4	medaka	LG3	36623554	2.00E-11	31113012	84
100566	5	medaka	LG4	34636364	4.00E-28	4980768	100
107800	5	medaka	LG4	34636364	1.00E-12	4330103	98
15733	5	medaka	LG4	34636364	1.00E-18	19572539	72
77318	5	medaka	LG2	31118443	2.00E-11	15975794	44
74916	6	medaka	scaffold1291	41275	8.00E-20	33765	82
75436	6	medaka	LG16	30014384	3.00E-13	9175586	79
34216	6	medaka	LG16	30014384	5.00E-21	24841705	68
38956	7	medaka	LG7	29492121	1.00E-12	10771070	42
27002	7	medaka	LG7	29492121	8.00E-14	7334160	84
97846	8	medaka	LG2	31118443	3.00E-13	14773235	87
68802	8	medaka	LG4	34636364	5.00E-12	16655998	53
94574	9	medaka	ultracontig200	430158	1.00E-21	59730	85
27881	10	medaka	LG17	31848461	4.00E-25	17111205	103
21526	10	medaka	LG21	31883787	2.00E-11	24961711	68
88334	11	medaka	LG10	27595823	8.00E-17	16770921	85
72849	13	medaka	LG22	28810691	3.00E-22	7337472	82
75998	13	medaka	scaffold683	122730	7.00E-11	5028	55
120000	13	medaka	LG22	28810691	8.00E-20	3630293	110
129708	14	medaka	LG4	34636364	5.00E-21	9010233	88
55683	15	medaka	LG17	31848461	2.00E-11	12931451	92
68027	15	medaka	LG5	33792114	1.00E-12	6695963	94
89023	17	medaka	LG24	24165179	1.00E-18	23791478	108
31725	18	medaka	LG4	34636364	1.00E-12	16656003	50
68597	18	medaka	LG1	39973033	2.00E-14	23062020	69
35783	18	medaka	LG8	25865442	5.00E-12	18836136	65
29586	18	medaka	LG11	29412213	2.00E-11	16782738	64
67084	19	medaka	LG17	31848461	1.00E-34	23372248	95
4370	21	medaka	LG23	24050845	5.00E-15	20523753	110
134952	21	medaka	scaffold860	79008	7.00E-11	55225	59
29868	22	medaka	LG4	34636364	3.00E-19	29433512	97
72264	23	medaka	LG2	31118443	7.00E-11	4404859	111
53364	23	medaka	LG7	29492121	2.00E-32	22947530	107
