# Supplementary figures and images for: A Hybrid Genetic Linkage Map of Two Ecologically and Morphologically Divergent Midas Cichlid Fishes (Amphilophus spp.) Obtained by Massively Parallel DNA Sequencing (ddRADSeq)
Source: G3 (Bethesda). 2013 Jan 1;3(1):65–74. doi: 10.1534/g3.112.003897 (PMC3538344; doi:10.1534/g3.112.003897)

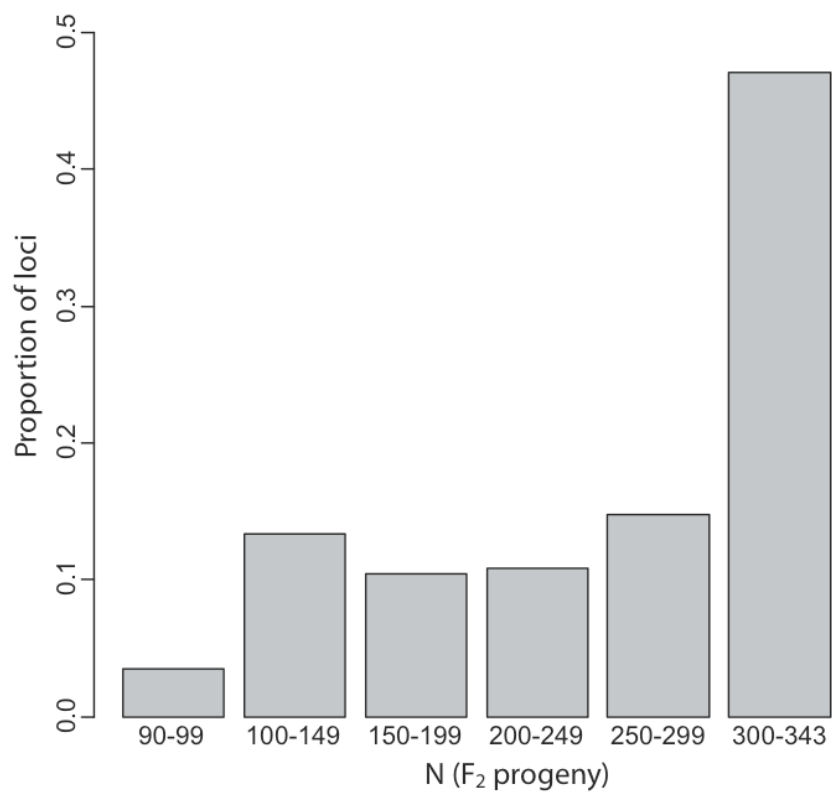

**Figure S1** Proportion of loci present in number (N) of individuals

Supplement: Supporting Information [file supp_3.1.65_FigureS1.pdf]
