# Supplementary material for: A Hybrid Genetic Linkage Map of Two Ecologically and Morphologically Divergent Midas Cichlid Fishes (Amphilophus spp.) Obtained by Massively Parallel DNA Sequencing (ddRADSeq)
Source: G3 (Bethesda). 2013 Jan 1;3(1):65–74. doi: 10.1534/g3.112.003897 (PMC3538344; doi:10.1534/g3.112.003897)
Supplement: Supporting Information [file supp_3.1.65_TableS1.pdf]

Table S1   P1 and P2 adapter sequences

ID	Sequence
KE_P1thioFwAACCC	AATGATACGGCGACCACCGAGATCTACACTCTTTCCCTACACGACGCTCTTCCGATCTAACCCTGCA
KE_P1thioFwAAGGG	AATGATACGGCGACCACCGAGATCTACACTCTTTCCCTACACGACGCTCTTCCGATCTAAGGGTGCA
KE_P1thioFwACTGC	AATGATACGGCGACCACCGAGATCTACACTCTTTCCCTACACGACGCTCTTCCGATCTACTGCTGCA
KE_P1thioFwAGAGT	AATGATACGGCGACCACCGAGATCTACACTCTTTCCCTACACGACGCTCTTCCGATCTAGAGTTGCA
KE_P1thioFwAGCTG	AATGATACGGCGACCACCGAGATCTACACTCTTTCCCTACACGACGCTCTTCCGATCTAGCTGTGCA
KE_P1thioFwAGGAC	AATGATACGGCGACCACCGAGATCTACACTCTTTCCCTACACGACGCTCTTCCGATCTAGGACTGCA
KE_P1thioFwAGTCA	AATGATACGGCGACCACCGAGATCTACACTCTTTCCCTACACGACGCTCTTCCGATCTAGTCATGCA
KE_P1thioFwATATC	AATGATACGGCGACCACCGAGATCTACACTCTTTCCCTACACGACGCTCTTCCGATCTATATCTGCA
KE_P1thioFwATCGA	AATGATACGGCGACCACCGAGATCTACACTCTTTCCCTACACGACGCTCTTCCGATCTATCGATGCA
KE_P1thioFwATGCT	AATGATACGGCGACCACCGAGATCTACACTCTTTCCCTACACGACGCTCTTCCGATCTATGCTTGCA
KE_P1thioFwATTAG	AATGATACGGCGACCACCGAGATCTACACTCTTTCCCTACACGACGCTCTTCCGATCTATTAGTGCA
KE_P1thioFwCAACT	AATGATACGGCGACCACCGAGATCTACACTCTTTCCCTACACGACGCTCTTCCGATCTCAACTTGCA
KE_P1thioFwCACAG	AATGATACGGCGACCACCGAGATCTACACTCTTTCCCTACACGACGCTCTTCCGATCTCACAGTGCA
KE_P1thioFwCAGTC	AATGATACGGCGACCACCGAGATCTACACTCTTTCCCTACACGACGCTCTTCCGATCTCAGTCTGCA
KE_P1thioFwCATGA	AATGATACGGCGACCACCGAGATCTACACTCTTTCCCTACACGACGCTCTTCCGATCTCATGATGCA
KE_P1thioFwCCAAC	AATGATACGGCGACCACCGAGATCTACACTCTTTCCCTACACGACGCTCTTCCGATCTCCAACTGCA
KE_P1thioFwCCCCA	AATGATACGGCGACCACCGAGATCTACACTCTTTCCCTACACGACGCTCTTCCGATCTCCCCATGCA
KE_P1thioFwCCGGT	AATGATACGGCGACCACCGAGATCTACACTCTTTCCCTACACGACGCTCTTCCGATCTCCGGTTGCA
KE_P1thioFwCCTTG	AATGATACGGCGACCACCGAGATCTACACTCTTTCCCTACACGACGCTCTTCCGATCTCCTTGTGCA
KE_P1thioFwCGATA	AATGATACGGCGACCACCGAGATCTACACTCTTTCCCTACACGACGCTCTTCCGATCTCGATATGCA
KE_P1thioFwCGCGC	AATGATACGGCGACCACCGAGATCTACACTCTTTCCCTACACGACGCTCTTCCGATCTCGCGCTGCA
KE_P1thioFwCGGCG	AATGATACGGCGACCACCGAGATCTACACTCTTTCCCTACACGACGCTCTTCCGATCTCGGCGTGCA
KE_P1thioFwCGTAT	AATGATACGGCGACCACCGAGATCTACACTCTTTCCCTACACGACGCTCTTCCGATCTCGTATTGCA
KE_P1thioFwCTAGG	AATGATACGGCGACCACCGAGATCTACACTCTTTCCCTACACGACGCTCTTCCGATCTCTAGGTGCA
KE_P1thioFwCTCTT	AATGATACGGCGACCACCGAGATCTACACTCTTTCCCTACACGACGCTCTTCCGATCTCTCTTTGCA
KE_P1thioFwCTTCC	AATGATACGGCGACCACCGAGATCTACACTCTTTCCCTACACGACGCTCTTCCGATCTCTTCCTGCA
KE_P1thioFwGAAGC	AATGATACGGCGACCACCGAGATCTACACTCTTTCCCTACACGACGCTCTTCCGATCTGAAGCTGCA
KE_P1thioFwGACTA	AATGATACGGCGACCACCGAGATCTACACTCTTTCCCTACACGACGCTCTTCCGATCTGACTATGCA
KE_P1thioFwGAGAT	AATGATACGGCGACCACCGAGATCTACACTCTTTCCCTACACGACGCTCTTCCGATCTGAGATTGCA
KE_P1thioFwGATCG	AATGATACGGCGACCACCGAGATCTACACTCTTTCCCTACACGACGCTCTTCCGATCTGATCGTGCA
KE_P1thioFwGCATT	AATGATACGGCGACCACCGAGATCTACACTCTTTCCCTACACGACGCTCTTCCGATCTGCATTTGCA
KE_P1thioFwGCCGG	AATGATACGGCGACCACCGAGATCTACACTCTTTCCCTACACGACGCTCTTCCGATCTGCCGGTGCA
KE_P1thioFwGCGCC	AATGATACGGCGACCACCGAGATCTACACTCTTTCCCTACACGACGCTCTTCCGATCTGCGCCTGCA
KE_P1thioFwGCTAA	AATGATACGGCGACCACCGAGATCTACACTCTTTCCCTACACGACGCTCTTCCGATCTGCTAATGCA
KE_P1thioFwGGAAG	AATGATACGGCGACCACCGAGATCTACACTCTTTCCCTACACGACGCTCTTCCGATCTGGAAGTGCA
KE_P1thioFwGGCCT	AATGATACGGCGACCACCGAGATCTACACTCTTTCCCTACACGACGCTCTTCCGATCTGGCCTTGCA
KE_P1thioFwGGGGA	AATGATACGGCGACCACCGAGATCTACACTCTTTCCCTACACGACGCTCTTCCGATCTGGGGATGCA
KE_P1thioFwGGTTC	AATGATACGGCGACCACCGAGATCTACACTCTTTCCCTACACGACGCTCTTCCGATCTGGTTCTGCA

KE_P1thioFwGTACA	AATGATACGGCGACCACCGAGATCTACACTCTTTCCCTACACGACGCTCTTCCGATCTGTACATGCA
KE_P1thioFwGTCAC	AATGATACGGCGACCACCGAGATCTACACTCTTTCCCTACACGACGCTCTTCCGATCTGTCACTGCA
KE_P1thioFwGTGTG	AATGATACGGCGACCACCGAGATCTACACTCTTTCCCTACACGACGCTCTTCCGATCTGTGTGTGCA
KE_P1thioFwGTTGT	AATGATACGGCGACCACCGAGATCTACACTCTTTCCCTACACGACGCTCTTCCGATCTGTTGTTGCA
KE_P1thioFwTTAAT	AATGATACGGCGACCACCGAGATCTACACTCTTTCCCTACACGACGCTCTTCCGATCTTTAATTGCA
KE_P1thioFwTTCCG	AATGATACGGCGACCACCGAGATCTACACTCTTTCCCTACACGACGCTCTTCCGATCTTTCCGTGCA
KE_P1thioFwAAAAA	AATGATACGGCGACCACCGAGATCTACACTCTTTCCCTACACGACGCTCTTCCGATCTAAAAATGCA
KE_P1thioFwAATTT	AATGATACGGCGACCACCGAGATCTACACTCTTTCCCTACACGACGCTCTTCCGATCTAATTTTGCA
KE_P1thioFwACACG	AATGATACGGCGACCACCGAGATCTACACTCTTTCCCTACACGACGCTCTTCCGATCTACACGTGCA
KE_P1thioFwACCAT	AATGATACGGCGACCACCGAGATCTACACTCTTTCCCTACACGACGCTCTTCCGATCTACCATTGCA
KE_P1thioFwACGTA	AATGATACGGCGACCACCGAGATCTACACTCTTTCCCTACACGACGCTCTTCCGATCTACGTATGCA
KE_P1thioFwTTTTT	AATGATACGGCGACCACCGAGATCTACACTCTTTCCCTACACGACGCTCTTCCGATCTTTTTTTGCA
KE_P1PthioRvAACCC	GGGTTAGATCGGAAGAGCGTCGTGTAGGGAAAGAGTGTAGATCTCGGTGGTCGCCGTATCATT
KE_P1PthioRvAAGGG	CCCTTAGATCGGAAGAGCGTCGTGTAGGGAAAGAGTGTAGATCTCGGTGGTCGCCGTATCATT
KE_P1PthioRvACTGC	GCAGTAGATCGGAAGAGCGTCGTGTAGGGAAAGAGTGTAGATCTCGGTGGTCGCCGTATCATT
KE_P1PthioRvAGAGT	ACTCTAGATCGGAAGAGCGTCGTGTAGGGAAAGAGTGTAGATCTCGGTGGTCGCCGTATCATT
KE_P1PthioRvAGCTG	CAGCTAGATCGGAAGAGCGTCGTGTAGGGAAAGAGTGTAGATCTCGGTGGTCGCCGTATCATT
KE_P1PthioRvAGGAC	GTCCTAGATCGGAAGAGCGTCGTGTAGGGAAAGAGTGTAGATCTCGGTGGTCGCCGTATCATT
KE_P1PthioRvAGTCA	TGACTAGATCGGAAGAGCGTCGTGTAGGGAAAGAGTGTAGATCTCGGTGGTCGCCGTATCATT
KE_P1PthioRvATATC	GATATAGATCGGAAGAGCGTCGTGTAGGGAAAGAGTGTAGATCTCGGTGGTCGCCGTATCATT
KE_P1PthioRvATCGA	TCGATAGATCGGAAGAGCGTCGTGTAGGGAAAGAGTGTAGATCTCGGTGGTCGCCGTATCATT
KE_P1PthioRvATGCT	AGCATAGATCGGAAGAGCGTCGTGTAGGGAAAGAGTGTAGATCTCGGTGGTCGCCGTATCATT
KE_P1PthioRvATTAG	CTAATAGATCGGAAGAGCGTCGTGTAGGGAAAGAGTGTAGATCTCGGTGGTCGCCGTATCATT
KE_P1PthioRvCAACT	AGTTGAGATCGGAAGAGCGTCGTGTAGGGAAAGAGTGTAGATCTCGGTGGTCGCCGTATCATT
KE_P1PthioRvCACAG	CTGTGAGATCGGAAGAGCGTCGTGTAGGGAAAGAGTGTAGATCTCGGTGGTCGCCGTATCATT
KE_P1PthioRvCAGTC	GACTGAGATCGGAAGAGCGTCGTGTAGGGAAAGAGTGTAGATCTCGGTGGTCGCCGTATCATT
KE_P1PthioRvCATGA	TCATGAGATCGGAAGAGCGTCGTGTAGGGAAAGAGTGTAGATCTCGGTGGTCGCCGTATCATT
KE_P1PthioRvCCAAC	GTTGGAGATCGGAAGAGCGTCGTGTAGGGAAAGAGTGTAGATCTCGGTGGTCGCCGTATCATT
KE_P1PthioRvCCCCA	TGGGGAGATCGGAAGAGCGTCGTGTAGGGAAAGAGTGTAGATCTCGGTGGTCGCCGTATCATT
KE_P1PthioRvCCGGT	ACCGGAGATCGGAAGAGCGTCGTGTAGGGAAAGAGTGTAGATCTCGGTGGTCGCCGTATCATT
KE_P1PthioRvCCTTG	CAAGGAGATCGGAAGAGCGTCGTGTAGGGAAAGAGTGTAGATCTCGGTGGTCGCCGTATCATT
KE_P1PthioRvCGATA	TATCGAGATCGGAAGAGCGTCGTGTAGGGAAAGAGTGTAGATCTCGGTGGTCGCCGTATCATT
KE_P1PthioRvCGCGC	GCGCGAGATCGGAAGAGCGTCGTGTAGGGAAAGAGTGTAGATCTCGGTGGTCGCCGTATCATT
KE_P1PthioRvCGGCG	CGCCGAGATCGGAAGAGCGTCGTGTAGGGAAAGAGTGTAGATCTCGGTGGTCGCCGTATCATT
KE_P1PthioRvCGTAT	ATACGAGATCGGAAGAGCGTCGTGTAGGGAAAGAGTGTAGATCTCGGTGGTCGCCGTATCATT
KE_P1PthioRvCTAGG	CCTAGAGATCGGAAGAGCGTCGTGTAGGGAAAGAGTGTAGATCTCGGTGGTCGCCGTATCATT
KE_P1PthioRvCTCTT	AAGAGAGATCGGAAGAGCGTCGTGTAGGGAAAGAGTGTAGATCTCGGTGGTCGCCGTATCATT
KE_P1PthioRvCTTCC	GGAAGAGATCGGAAGAGCGTCGTGTAGGGAAAGAGTGTAGATCTCGGTGGTCGCCGTATCATT
KE_P1PthioRvGAAGC	GCTTCAGATCGGAAGAGCGTCGTGTAGGGAAAGAGTGTAGATCTCGGTGGTCGCCGTATCATT
KE_P1PthioRvGACTA	TAGTCAGATCGGAAGAGCGTCGTGTAGGGAAAGAGTGTAGATCTCGGTGGTCGCCGTATCATT

KE_P1PthioRvGAGAT	ATCTCAGATCGGAAGAGCGTCGTGTAGGGAAAGAGTGTAGATCTCGGTGGTCGCCGTATCATT
KE_P1PthioRvGATCG	CGATCAGATCGGAAGAGCGTCGTGTAGGGAAAGAGTGTAGATCTCGGTGGTCGCCGTATCATT
KE_P1PthioRvGCATT	AATGCAGATCGGAAGAGCGTCGTGTAGGGAAAGAGTGTAGATCTCGGTGGTCGCCGTATCATT
KE_P1PthioRvGCCGG	CCGGCAGATCGGAAGAGCGTCGTGTAGGGAAAGAGTGTAGATCTCGGTGGTCGCCGTATCATT
KE_P1PthioRvGCGCC	GGCGCAGATCGGAAGAGCGTCGTGTAGGGAAAGAGTGTAGATCTCGGTGGTCGCCGTATCATT
KE_P1PthioRvGCTAA	TTAGCAGATCGGAAGAGCGTCGTGTAGGGAAAGAGTGTAGATCTCGGTGGTCGCCGTATCATT
KE_P1PthioRvGGAAG	CTTCCAGATCGGAAGAGCGTCGTGTAGGGAAAGAGTGTAGATCTCGGTGGTCGCCGTATCATT
KE_P1PthioRvGGCCT	AGGCCAGATCGGAAGAGCGTCGTGTAGGGAAAGAGTGTAGATCTCGGTGGTCGCCGTATCATT
KE_P1PthioRvGGGGA	TCCCCAGATCGGAAGAGCGTCGTGTAGGGAAAGAGTGTAGATCTCGGTGGTCGCCGTATCATT
KE_P1PthioRvGGTTC	GAACCAGATCGGAAGAGCGTCGTGTAGGGAAAGAGTGTAGATCTCGGTGGTCGCCGTATCATT
KE_P1PthioRvGTACA	TGTACAGATCGGAAGAGCGTCGTGTAGGGAAAGAGTGTAGATCTCGGTGGTCGCCGTATCATT
KE_P1PthioRvGTCAC	GTGACAGATCGGAAGAGCGTCGTGTAGGGAAAGAGTGTAGATCTCGGTGGTCGCCGTATCATT
KE_P1PthioRvGTGTG	CACACAGATCGGAAGAGCGTCGTGTAGGGAAAGAGTGTAGATCTCGGTGGTCGCCGTATCATT
KE_P1PthioRvGTTGT	ACAACAGATCGGAAGAGCGTCGTGTAGGGAAAGAGTGTAGATCTCGGTGGTCGCCGTATCATT
KE_P1PthioRvTTAAT	ATTAAAGATCGGAAGAGCGTCGTGTAGGGAAAGAGTGTAGATCTCGGTGGTCGCCGTATCATT
KE_P1PthioRvTTCCG	CGGAAAGATCGGAAGAGCGTCGTGTAGGGAAAGAGTGTAGATCTCGGTGGTCGCCGTATCATT
KE_P1PthioRvAAAAA	TTTTTAGATCGGAAGAGCGTCGTGTAGGGAAAGAGTGTAGATCTCGGTGGTCGCCGTATCATT
KE_P1PthioRvAATTT	AAATTAGATCGGAAGAGCGTCGTGTAGGGAAAGAGTGTAGATCTCGGTGGTCGCCGTATCATT
KE_P1PthioRvACACG	CGTGTAGATCGGAAGAGCGTCGTGTAGGGAAAGAGTGTAGATCTCGGTGGTCGCCGTATCATT
KE_P1PthioRvACCAT	ATGGTAGATCGGAAGAGCGTCGTGTAGGGAAAGAGTGTAGATCTCGGTGGTCGCCGTATCATT
KE_P1PthioRvACGTA	TACGTAGATCGGAAGAGCGTCGTGTAGGGAAAGAGTGTAGATCTCGGTGGTCGCCGTATCATT
KE_P1PthioRvTTTTT	AAAAAAGATCGGAAGAGCGTCGTGTAGGGAAAGAGTGTAGATCTCGGTGGTCGCCGTATCATT
KE_P2PhoFwCGpair	CGAGATCGGAAGAGCGGTTCAGCAGGAATGCCGAGACCGATCAGAACAA
KE_P2thioRvpair	CAAGCAGAAGACGGCATACGAGATCGGTCTCGGCATTCCTGCTGAACCGCTCTTCCGATCT

Identification (=ID) defines adapter type (P1 or P2), modification (phosphorothioate bond = thio, 5' Phosphorylation = P), direction (Fw = forward, RV = reverse), and 5 base barcode.
