# Supplementary material for: A Hybrid Genetic Linkage Map of Two Ecologically and Morphologically Divergent Midas Cichlid Fishes (Amphilophus spp.) Obtained by Massively Parallel DNA Sequencing (ddRADSeq)
Source: G3 (Bethesda). 2013 Jan 1;3(1):65–74. doi: 10.1534/g3.112.003897 (PMC3538344; doi:10.1534/g3.112.003897)
Supplement: Supporting Information [file supp_3.1.65_TableS2.pdf]

Table S2   Individual's sequenced genomic output

	N	N loci	N loci polymorphic	N SNPs	SNP frequency	coverage
A. zaliosus, male	1	142740	9531	12764	0.089	15.6x
A. astorquii, female	1	139109	8150	11172	0.080	15.0x
F1 hybrids	2	140925	9336	11968	0.090	14.0x
F2 hybrids	343	36851	1633	2257	0.061	15.2x

Note that in the parents and F_1 hybrids a higher gel size range was selected than for F_2 individuals and therefore they exhibit a higher number of loci.
