# Supplementary material for: A Hybrid Genetic Linkage Map of Two Ecologically and Morphologically Divergent Midas Cichlid Fishes (Amphilophus spp.) Obtained by Massively Parallel DNA Sequencing (ddRADSeq)
Source: G3 (Bethesda). 2013 Jan 1;3(1):65–74. doi: 10.1534/g3.112.003897 (PMC3538344; doi:10.1534/g3.112.003897)
Supplement: Supporting Information [file supp_3.1.65_TableS4.pdf]

Table S4 Length and number of markers per linkage group and average spacing distance between markers

LG	Spacing [cM]	Markers [N]	Length [cM]
1	1.61	49	77.1
2	2.56	28	69.2
3	2.16	33	69.1
4	1.44	49	69.0
5	1.25	54	66.1
6	2.18	31	65.4
7	3.27	21	65.4
8	2.84	24	65.3
9	3.32	20	63.0
10	1.53	42	62.9
11	1.67	38	61.9
12	2.18	29	61.1
13	1.89	33	60.4
14	2.99	21	59.8
15	1.42	43	59.7
16	4.57	14	59.4
17	1.36	44	58.4
18	1.87	32	58.0
19	2.04	29	57.1
20	6.07	10	54.6
21	1.65	34	54.4
22	2.69	21	53.7
23	1.03	51	51.5
24	1.27	3	2.5
25	1.99	2	2.0
**Average**	1.95	30.2	59.4
**Total**	-	755	1426.9
